# Combined morpho-physiological, ionomic and transcriptomic analyses reveal adaptive responses of allohexaploid wheat (*Triticum aestivum* L.) to iron deficiency

**DOI:** 10.1186/s12870-022-03627-4

**Published:** 2022-05-10

**Authors:** Ying-peng Hua, Yue Wang, Ting Zhou, Jin-yong Huang, Cai-peng Yue

**Affiliations:** School of Agricultural Sciences, Zhengzhou University, Zhengzhou, 450001 China

**Keywords:** Ion homeostasis, Low Fe stress, Morpho-physiological responses, Transcriptomic, *Triticum aestivum* L

## Abstract

**Background:**

Plants worldwide are often stressed by low Fe availability around the world, especially in aerobic soils. Therefore, the plant growth, seed yield, and quality of crop species are severely inhibited under Fe deficiency. Fe metabolism in plants is controlled by a series of complex transport, storage, and regulatory mechanisms in cells. Allohexaploid wheat (*Triticum aestivum* L.) is a staple upland crop species that is highly sensitive to low Fe stresses. Although some studies have been previously conducted on the responses of wheat plants to Fe deficiency, the key mechanisms underlying adaptive responses are still unclear in wheat due to its large and complex genome.

**Results:**

Transmission electron microscopy showed that the chloroplast structure was severely damaged under Fe deficiency. Paraffin sectioning revealed that the division rates of meristematic cells were reduced, and the sizes of elongated cells were diminished. ICP-MS-assisted ionmics analysis showed that low-Fe stress significantly limited the absorption of nutrients, including N, P, K, Ca, Mg, Fe, Mn, Cu, Zn, and B nutrients. High-throughput transcriptome sequencing identified 378 and 2,619 genome-wide differentially expressed genes (DEGs) were identified in the shoots and roots between high-Fe and low-Fe conditions, respectively. These DEGs were mainly involved in the Fe chelator biosynthesis, ion transport, photosynthesis, amino acid metabolism, and protein synthesis. Gene coexpression network diagrams indicated that *TaIRT1b-4A*, *TaNAS2-6D*, *TaNAS1a-6A*, *TaNAS1-6B,* and *TaNAAT1b-1D* might function as key regulators in the adaptive responses of wheat plants to Fe deficiency.

**Conclusions:**

These results might help us fully understand the morpho-physiological and molecular responses of wheat plants to low-Fe stress, and provide elite genetic resources for the genetic modification of efficient Fe use.

**Supplementary Information:**

The online version contains supplementary material available at 10.1186/s12870-022-03627-4.

## Background

Iron (Fe) functions as an essential micronutrient in maintaining the normal growth and development of higher plants, and it plays important roles in chlorophyll biosynthesis, respiration, redox reactions, and electron transfer [1]. Although the Fe content in soils is very high, it often exists in the form of hardly soluble Fe^3+^, which is hardly soluble in water due to the influence of soil pH and oxygen partial pressure [1, 2]. Therefore, Fe deficiency is more severe especially in calcareous soils with higher pH values [3]. Previous studies have shown that low Fe availability will not only inhibit the growth and development of plants but also affect the intake of essential Fe nutrients by animals and humans [2]. According to investigations, more than 40% of the world’s soils are severely Fe deficient [4]. Therefore, improving Fe use efficiency in plants has become a universal concern around the world. It has also been a popular tropic in plant nutrient studies since the beginning of this century.

Faced with different Fe nutritional statuses in the environment, higher plants have evolved two different strategies to adapt themselves to external Fe changes: a reduction strategy (Strategy I) and a chelation strategy (Strategy II) [4]. Monocotyledonous nongraminaceous plants and all dicotyledonous plants adopt Strategy I to deal with a low-Fe environment. When these plants lack Fe, their root epidermis adopts three processes of acidification, reduction, and absorption to absorb rhizosphere Fe [3]. First, a proton ATPase (H^+^-ATPase) is expressed in large quantities, pumping a large number of protons (H^+^) to acidify the rhizosphere environment, thereby improving the rhizosphere Fe availability. Second, the expression of *Fe reductase (FRO)* genes is upregulated, and then the Fe^3+^ adsorbed on the root epidermis is reduced to Fe^2+^. The expression of *iron-regulated transporter (IRT)* genes involved in the transport of Fe^2+^ into root cells is significantly induced by low Fe stress [3]. Strategy II is adopted by monocotyledonous gramineous plants and consists of two parts [5]. First, a plant Fe carrier (phytosiderophore, PS) binds to Fe^3+^ with high affinity, then it is secreted into the rhizosphere and combines with Fe^3+^ to form a Fe^3+^-PS complex. Under the action of the YSL (YS1-like protein) family transporters, the Fe^3+^-PS complex near the root epidermal cells is absorbed into monocotyledonous grasses [6]. The elucidation of Strategy I and Strategy II enables scientists to have a deeper understanding of the mechanism underlying the adaptive responses of plants to low Fe stresses, and also provides an important theoretical basis for solving the problem of low Fe use efficiency in plants.

Wheat (*Triticum aestivum* L.) is one of the most important upland crop species in the world [7]. Its seed yield and powder quality directly affect the improvement of human diets. Although many scholars have studied wheat roots under low Fe stress, most of them mainly focus on photosynthetic characteristics, root characteristics, seed germination, and plant physiological and biochemical effects [8–13]. There is still a lack of in-depth studies on the adaptability of wheat plants to low Fe stress. Therefore, studying the adaptive mechanism of wheat under low Fe stress has very important theoretical and practical guiding significance for improving the quality of crops.

The nutritional quality of crops, especially the effect of micronutrients such as Fe on human health, has attracted increasing attention. In recent years, important progress has been made in understanding the molecular mechanisms underlying Fe absorption, transport, and metabolism in higher plants. Increasing grain Fe contents in the grain has become a popular tropic for future enhanced biological breeding in the future. Revealing the molecular mechanisms underlying the efficient absorption and utilization of Fe in crop species, including wheat, has become an important direction for future plant nutrition research. Therefore, in this study, combined morpho-physiological, ionomic and transcriptomic analyses were performed to characterize key factors involved in the adaptive responses and use efficiency of Fe nutrients in allohexaploid wheat. This study is expected to provide a theoretical basis and elite gene resources for the breeding of new high-quality new varieties of wheat with high-efficiency Fe utilization.

## Results

### Morphological responses of wheat plants to low Fe stress

**Fig. 1:**
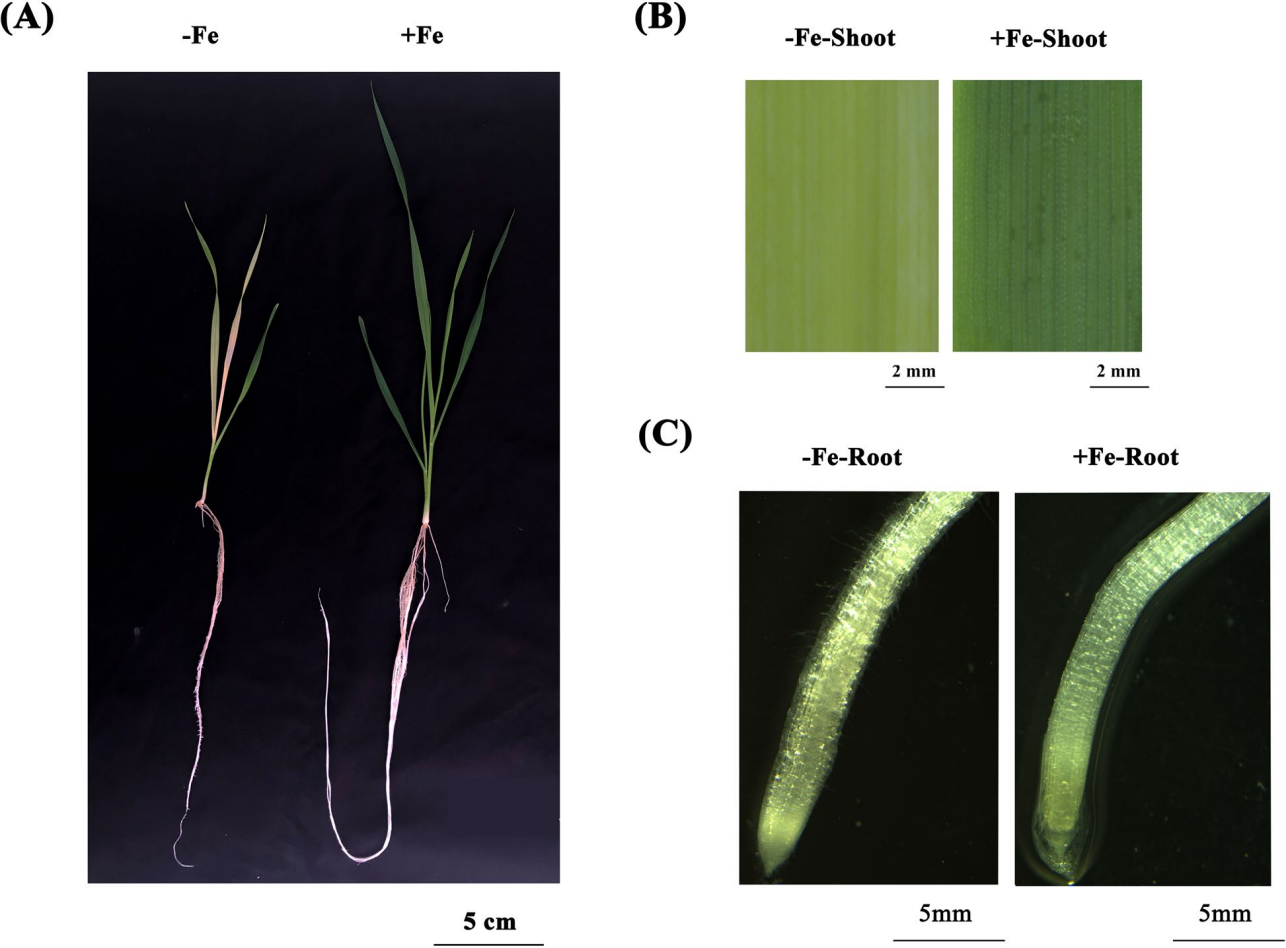
Morphological responses of wheat plants to low Fe stress. (**A**) Overall growth performance of wheat plants under high Fe (50 μM) and low Fe (2 μM) conditions for 10 days. Bar: 5 cm. **B-C** Leaf (**B**) and root (**C**) performance of wheat plants treated with different Fe concentrations. Bar: 2 mm

Our results showed that the root growth and development of wheat plants were inhibited under low Fe stress, whereas the root hair density increased (Fig. 1A, C). Young leaves were obviously chlorotic, and there was obviously visible intervein yellowing on the leaves (Fig. 1A, B).

**Fig. 2:**
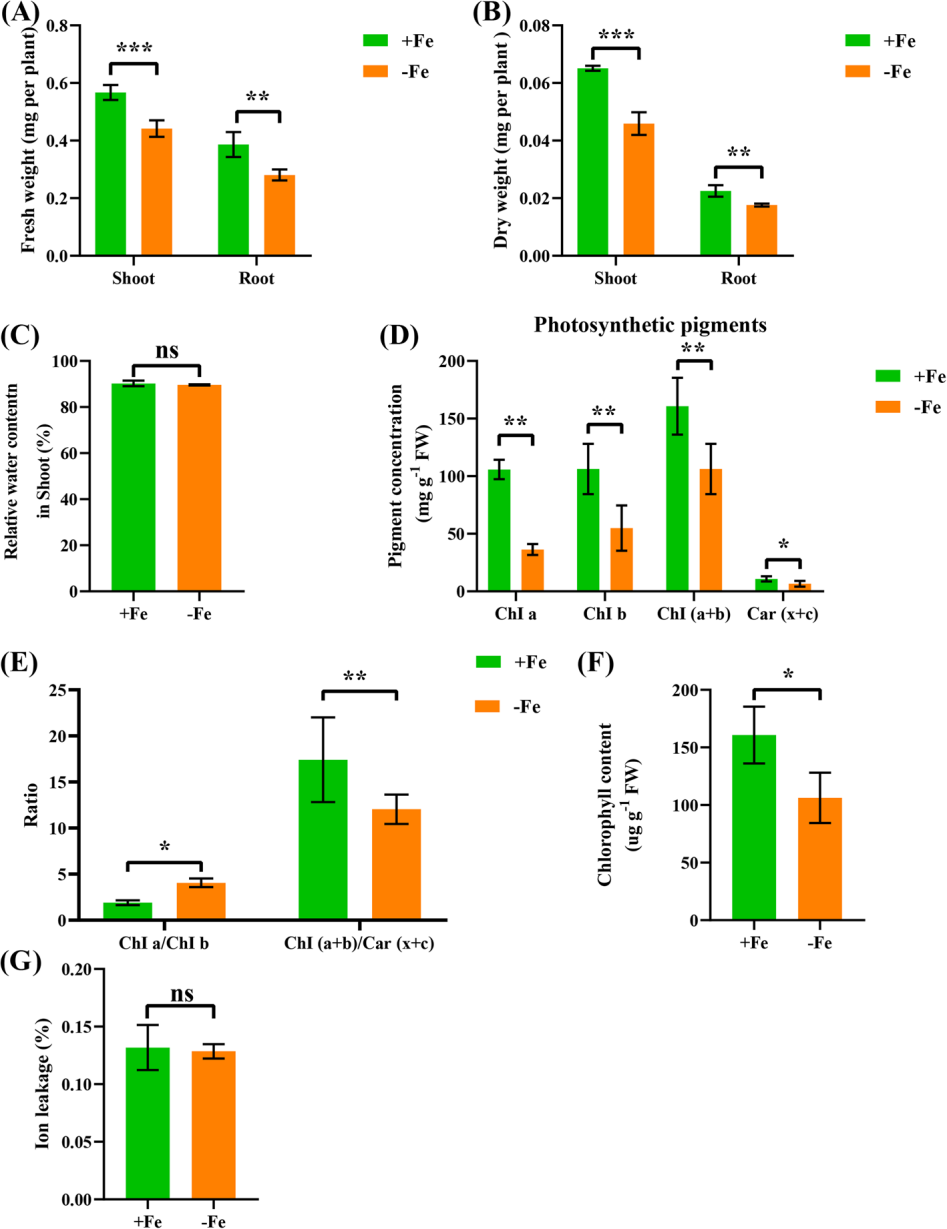
Physiological response of wheat to low Fe stress. (**A**) fresh weight, (**B**) dry weight, (**C**) relative water content (%) in the shoots, (**D**) pigment concentration, (**E**) ratio of Chl a/ChI b and Chl (a + b)/Chl (x + c), (**F**) chlorophyll concentration, (**G**) ion leakage. Data are means (± SD), *n* = 5. Significant differences (*, *P* < 0.05; **, *P* < 0.01; ***, *P* < 0.001) were determined by unpaired two-tailed Student’s *t*-test using Statistical Productions and Service Solutions 17.0 (SPSS, Chicago, IL, USA).

Under low Fe, the fresh and dry weights of the shoots decreased by approximately 22.1% and 19.3%, respectively; however, the fresh and dry weights of roots decreased by about 27.4% and 27.9%, respectively (Fig. 2A, B). The relative water content in the shoots showed no significant differences between the different Fe treatments (Fig. 2C). Under low Fe stress, the concentrations of total chlorophyll (including chlorophyll a and b) and carotenoids (including xanthophyll and carotene) were significantly reduced (Fig. 2D, F). The ratio of the concentration of chlorophyll a to chlorophyll b, a sign of plant adaptation to adversity stress, increased significantly under low Fe stress (Fig. 2E). In addition, low Fe stress reduced the ratio of the total chlorophyll concentration to the carotenoid concentration (Fig. 2E). We also measured the root ion leakage; however, we did not identify significant differences in the ion leakage rates between the different Fe treatments (Fig. 2G).

**Fig. 3:**
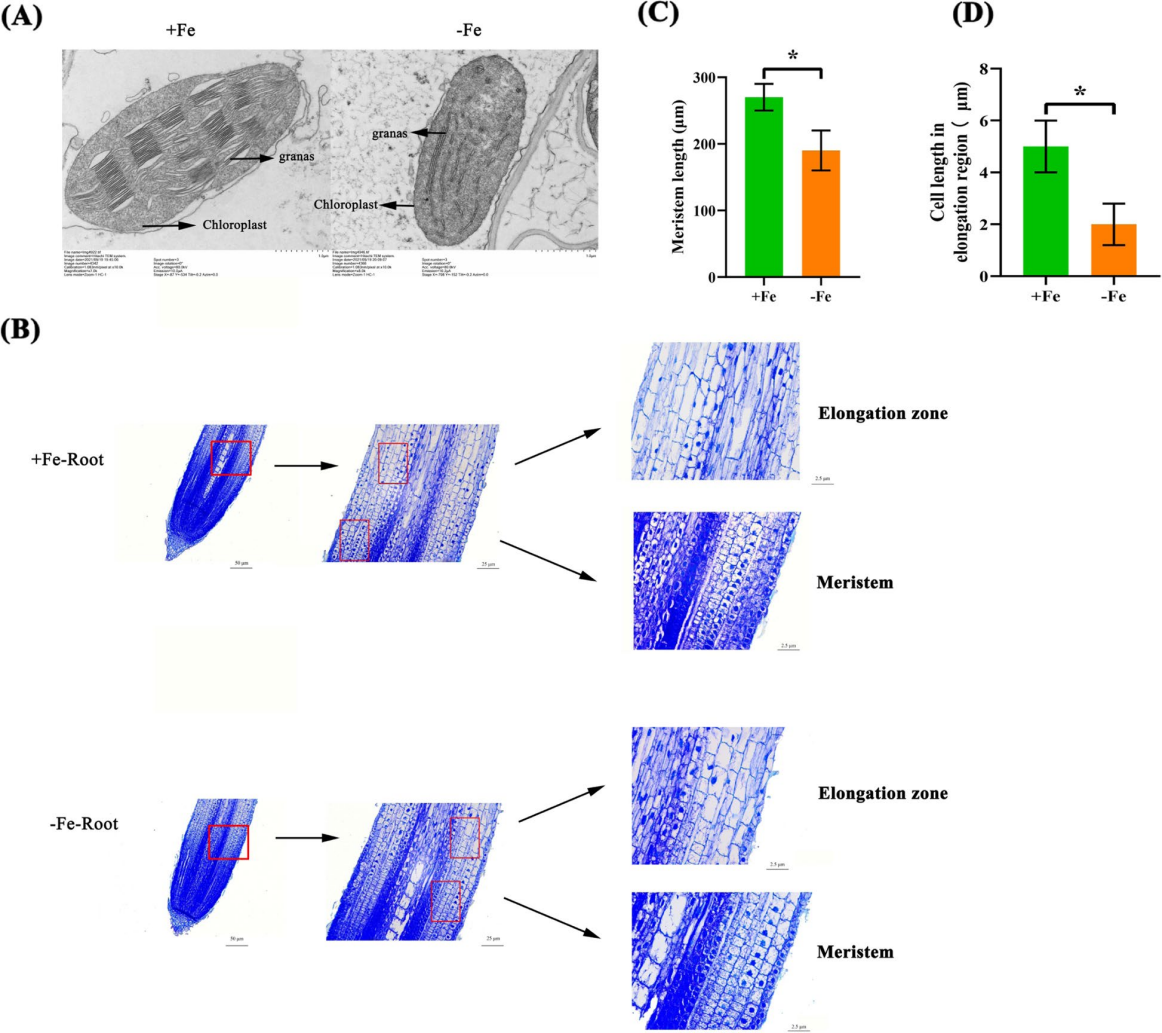
Microscopy analysis of wheat plant ultrastructure under low Fe stress. (**A**) Chloroplast structure, (**B**) effect of low Fe stress on the cells in the meristem and elongation zone of wheat roots. (**C**) length of the meristem; (**D**) cell length in elongation region. Significant differences (*, *P* < 0.05; **, *P* < 0.01; ***, *P* < 0.001) were determined by unpaired two-tailed Student’s *t*-test using Statistical Productions and Service Solutions 17.0 (SPSS, Chicago, IL, USA)

The intracellular ultrastructure of the morphological differences between the control and Fe-deficiency stress were examined by a transmission electron microscope (Fig. 3A). Our results showed that under Fe sufficiency, the chloroplasts were well developed, complete in structure, and had a convex lens shaped. Moreover, clear thylakoids, complete basal lamella, and multiple basal lamellas were orderly arranged. However, under low Fe stress, the chloroplast became severely deformed, and the lamellar system could not stack to form basal granules.

Root growth is determined by the balance between cell division and cell elongation [14]. To study the role of Fe in the growth and development of the root system architecture, we investigated the cell sizes of the meristematic zone and elongation zone in the roots of wheat plants grown under normal or low Fe stress (Fig. 3B). The meristematic zone is defined as the region of isodiametric cells from the quiescent centre (QC) up to the cell that is twice the length of the immediately preceding cell [15]. The boundary of the transition zone is different in each cell type; therefore, in all the analyses performed here, the cortical cell file is used to define the boundary [14]. Our results showed that compared with the control, the number of cells in the root meristematic zones under Fe-deficient conditions was significantly reduced, and the length of the cells in the elongation zones was also significantly diminished (Fig. 3B-D).

**Fig. 4:**
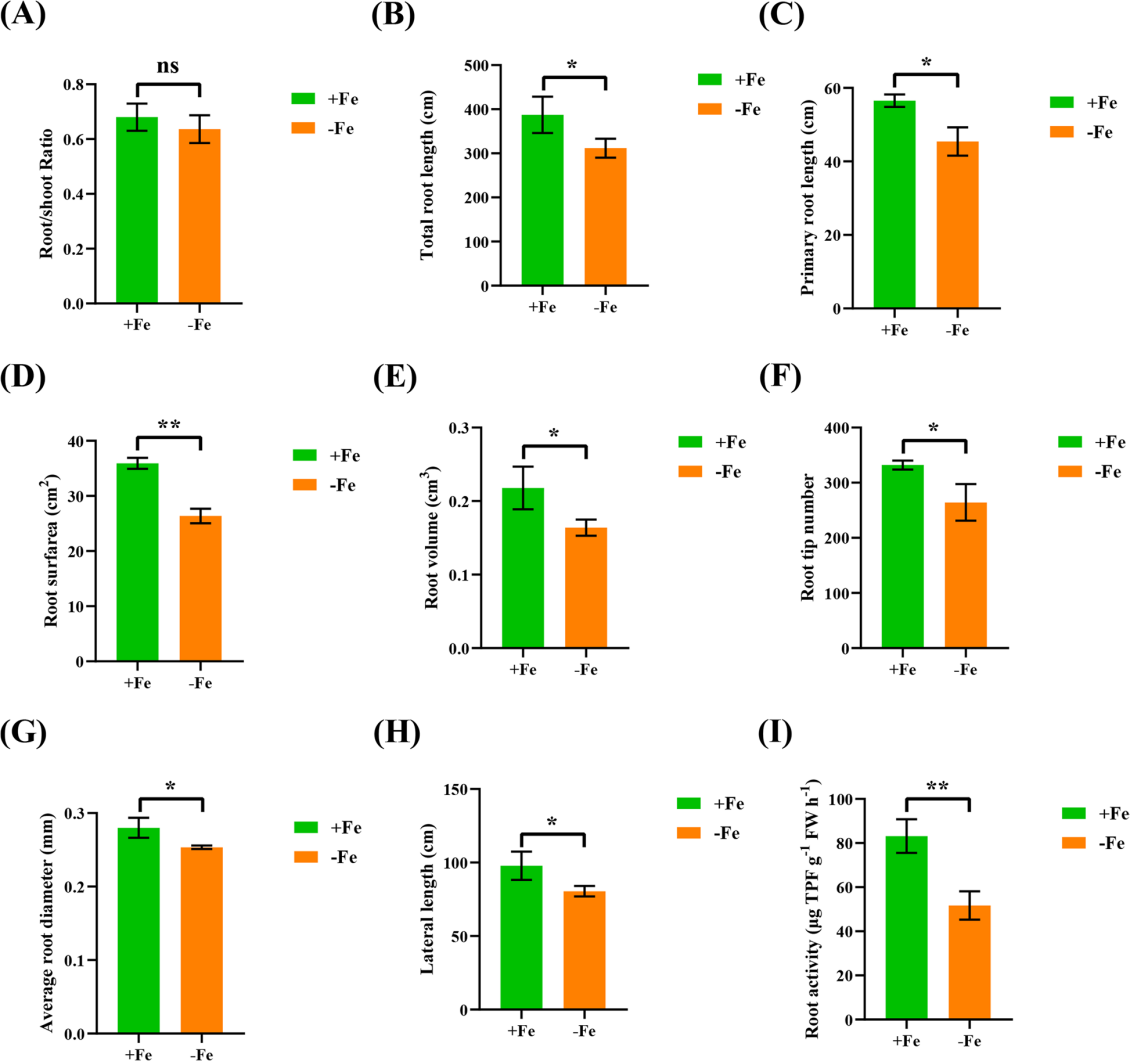
Effect of low Fe stress on root growth of wheat plants. (**A**) Root/shoot ratios, (**B**) total root length (cm), (**C**) primary root length (cm), (**D**) root surface (cm^2^), (**E**) root volume (cm^3^), (**F**) root tip numbers, (**G**) average root diameter (mm), (**H**) lateral length (cm), (**I**) root activity (μg TPF g^−1^ FW h^−1^). Data are means (± SD), *n* = 5. Significant differences (*, *P* < 0.05; **, *P* < 0.01; ***, *P* < 0.001) were determined by unpaired two-tailed Student’s *t*-test using Statistical Productions and Service Solutions 17.0 (SPSS, Chicago, IL, USA)

Under low Fe, the root-to-shoot ratio was reduced, whereas the difference was not significant (Fig. 4A). Subsequently, the specific effects of low Fe stress on root growth were analysed in wheat plants under high Fe and low Fe conditions. Low Fe stress significantly reduced root-related parameters, including total root length, maximal root length, root surface area, root volume, root tip number, average root diameter, and lateral root length (Fig. 4B-H). Then, we determined the root activity of wheat plants and found that the root activity of wheat seedlings was decreased significantly under low Fe stress (Fig. 4I). These results indicated that the growth and development of wheat seedlings were severely inhibited under low Fe stress.

### Physiological responses of wheat plants to low Fe

**Fig. 5:**
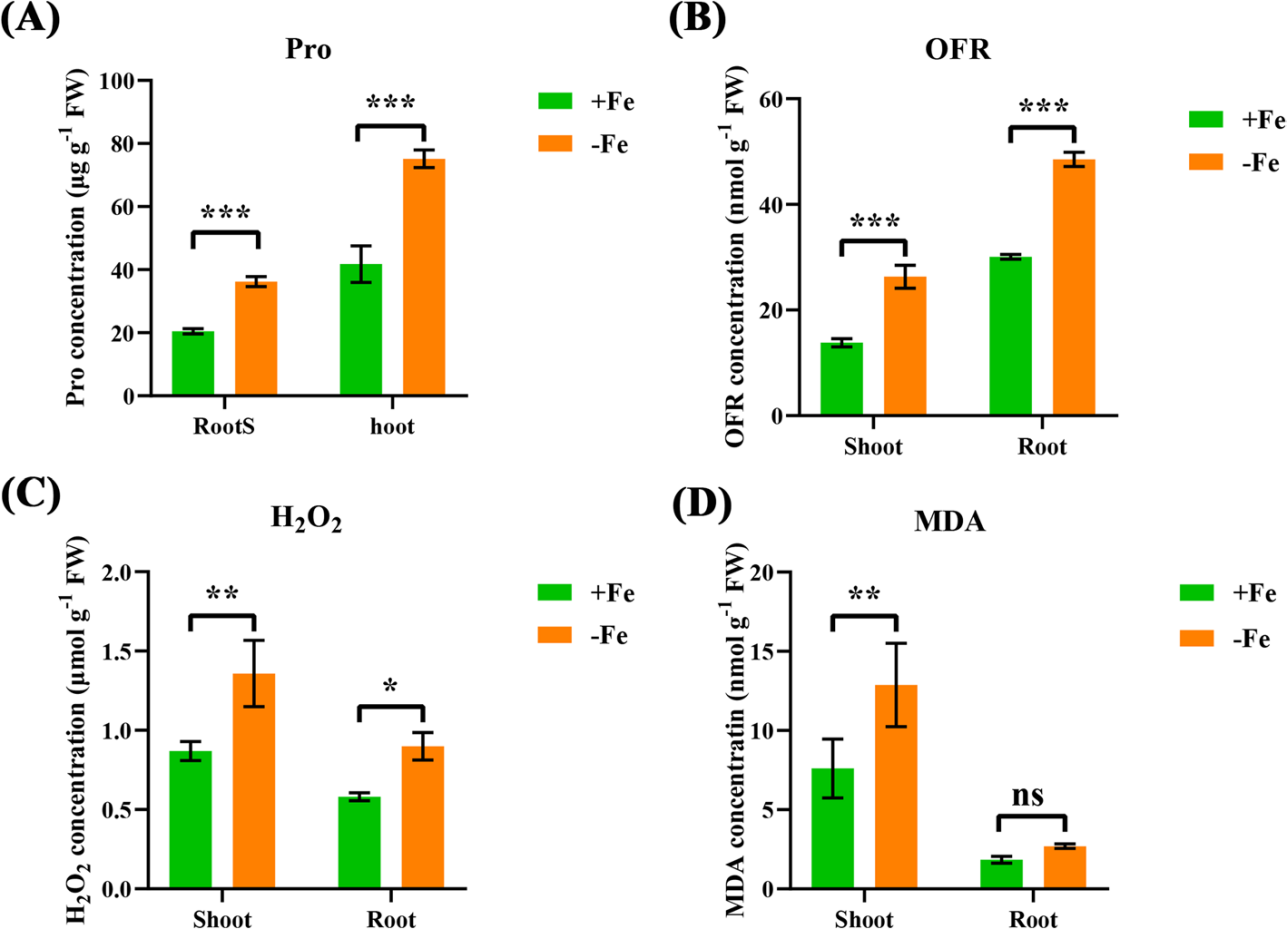
Effect of low Fe stress on osmotic adjustment substances in wheat. (**A**) proline (Pro), (**B**)superoxide anion (OFR), (**C**) hydrogen peroxide (H_2_O_2_), (**D**) malondialdehyde (MDA). Data are means (± SD), *n* = 5. Significant differences (*, *P* < 0.05; **, *P* < 0.01; ***, *P* < 0.001) were determined by unpaired two-tailed Student’s *t*-test using Statistical Productions and Service Solutions 17.0 (SPSS, Chicago, IL, USA)

To further understand the physiological responses of wheat plants to low Fe stress, we tested the contents of some osmotic adjustment substances that may participate in the regulation of low Fe resistance. Under low Fe stress, the proline (Pro) concentrations were significantly increased in both shoots and roots. (Fig. 5A, D). MDA is a sign of plasma membrane peroxidation. Our results showed that compared with the control, there was no significant difference in the concentration of MDA in the roots, although the MDA concentration increased significantly in the shoots under low Fe stress (Fig. 5D).

**Fig. 6:**
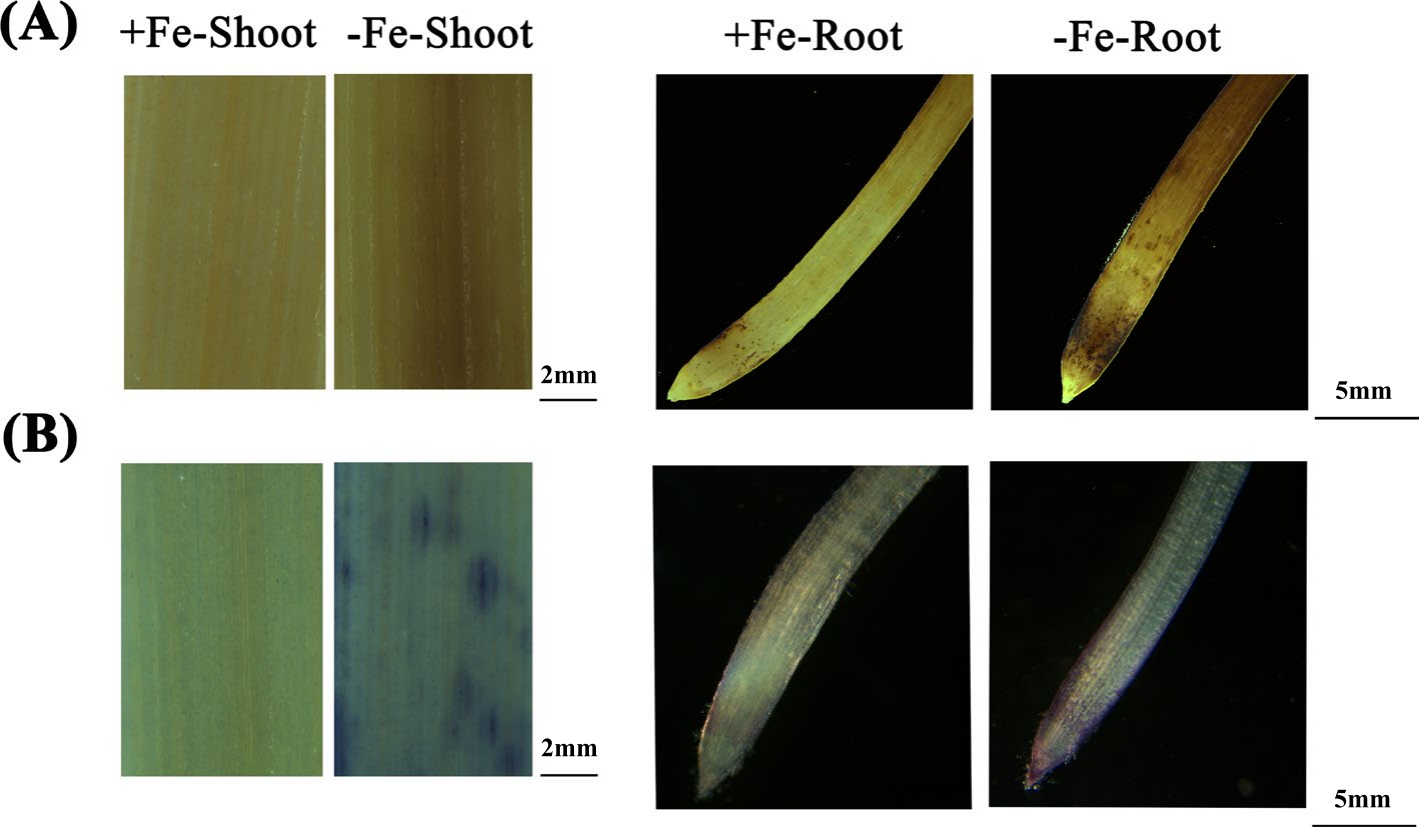
Effect of low Fe stress on ROS accumulation in wheat. **A-B** H_2_O_2_ (**A**) and O^2−^ (**B**) accumulation in wheat shoots and roots. The scale bar in the shoots is 2 mm, and the scale bar in the roots is 5 mm

When plants are stressed by Fe deficiency, they produce a large amount of ROS, which causes oxidative damage. The concentrations of OFR and H_2_O_2_ in boththe shoots and roots were significantly higher under low Fe than under the control condition (Fig. 5B, C). To confirm the effect of low Fe on the accumulation of ROS (H_2_O_2_ and O_2_^¯^) in wheat plants, we performed DAB and NBT staining on the shoots and roots of wheat plants after different Fe treatments (Fig. 6A, B). The results showed darker DAB and NBT staining of the shoots and roots of wheat plants under low Fe stress, which confirmed that more H_2_O_2_ and O_2_^¯^ accumulated in wheat plants under low Fe stress (Fig. 6A, B).

### Ionomic responses of wheat plants to low Fe

**Fig. 7:**
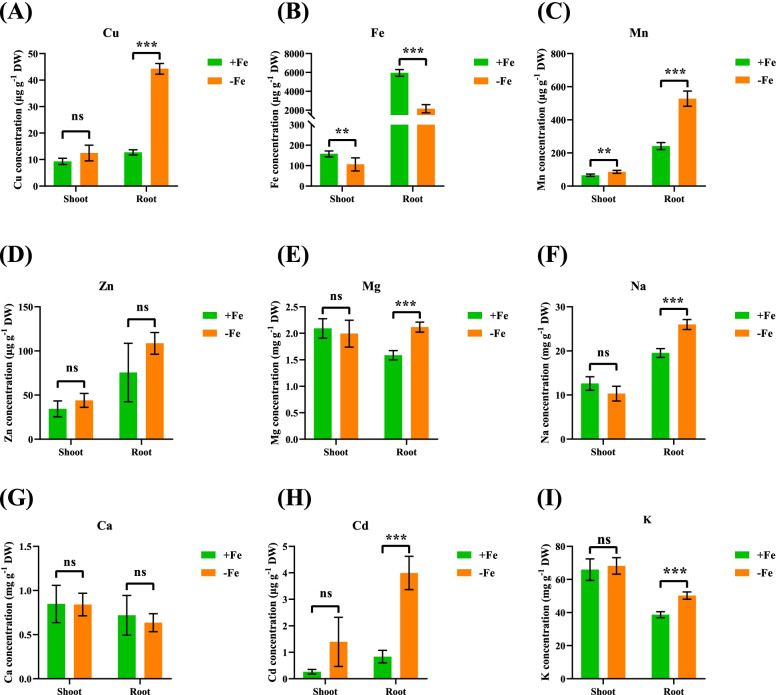
Ionomic analysis of wheat plants under different Fe treatments. (**A**) Cu, copper; (**B**) Fe, iron; (**C**) Mn, manganese; (**D**) Zn, zinc; (**E**) Mg, magnesium; (**F**) Na, sodium; (**G**) Ca, calcium; (**H**) Cd, cadmium; (**I**) K, potassium. Data are means (± SD), *n* = 5. Significant differences (*, *P* < 0.05; **, *P* < 0.01; ***, *P* < 0.001) were determined by unpaired two-tailed Student’s *t*-test using Statistical Productions and Service Solutions 17.0 (SPSS, Chicago, IL, USA)

ICP-MS quantitative analysis showed that the Fe^2+^ concentrations in the shoots and roots of wheat plants were significantly reduced under low Fe (Fig. 7B). We also tested the concentrations of other metal cations, including Cu^2+^, Mn^2+^, Zn^2+^, Mg^2+^, Na^+^, Ca^2+^, Cd^2+^, and K^+^ (Fig. 7). In general, the ion profiles of Cu^2+^, Mg^2+^, Na^+^, Cd^2+^, and K^+^ were similar under low Fe stress. The concentrations of Cu^2+^, Mg^2+^, Na^+^, Cd^2+^, and K^+^ in the shoots did not change significantly under low Fe stress; in contrast, their concentrations increased significantly in the roots (Fig. 7A, E, F, H, I). However, under low Fe stress, the Mn^2+^ concentrations increased significantly in both shoots and roots (Fig. 7C). In addition, the concentrations of both Zn^2+^ and Ca^2+^ did not change significantly in the shoots and roots (Fig. 7D, G).

### Genome-wide overview of the transcriptional responses of wheat plants to low Fe

**Fig. 8:**
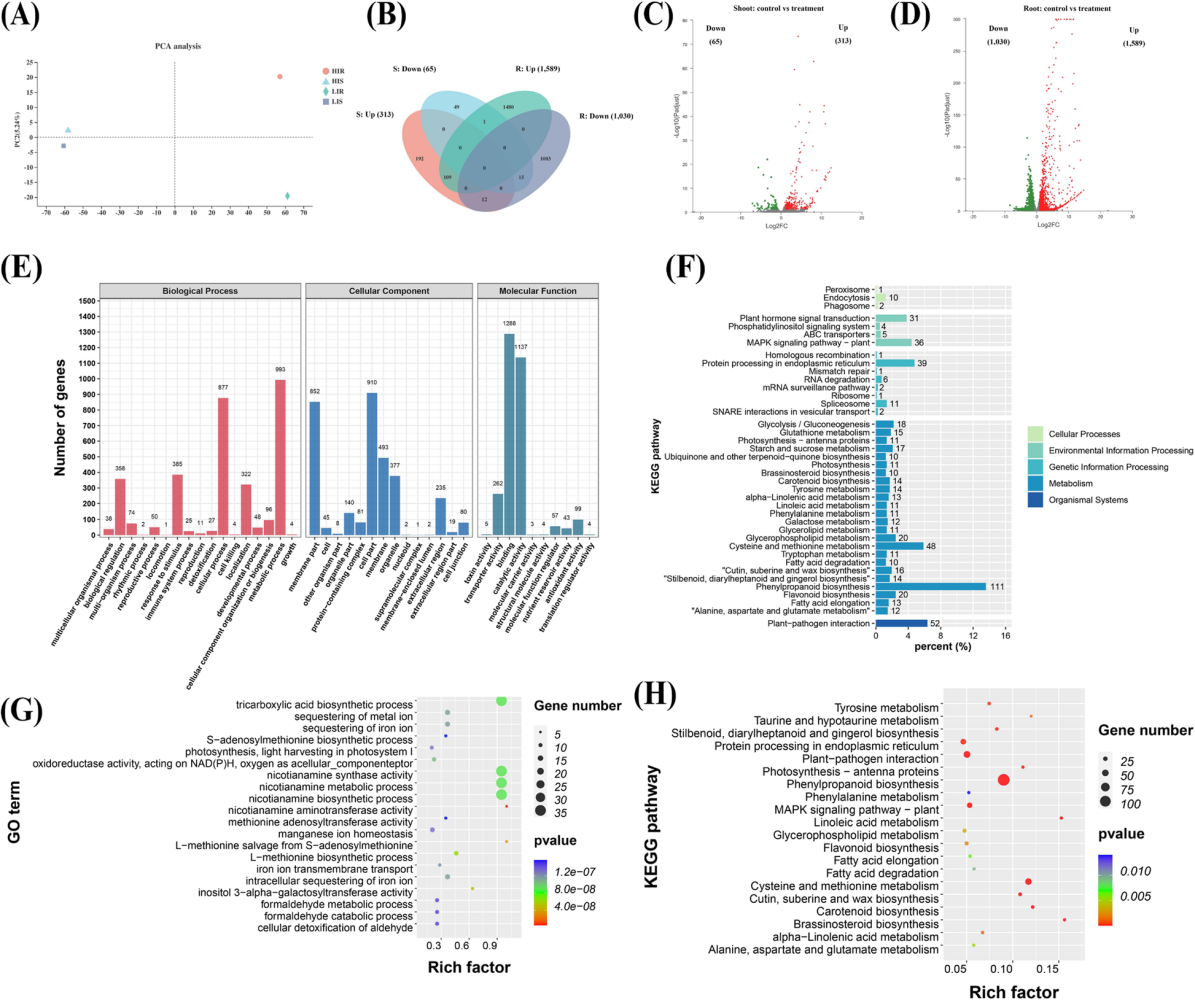
Transcriptomic analysis of wheat plants under different Fe treatments. (**A**) principal component analysis of differentially expressed genes (DEGs), (**B**) Venn diagram analysis, (**C-D**) volcano diagrams of DEGs in the shoots (**C**) and roots (**D**) under the control (normal Fe: 50 μM) and treatment (low Fe: 2 μM). GO annotation (**E**), KEGG pathway (**F**), GO enrichment (**G**) and KEGG enrichment (**H**) analysis of all DEGs in the shoots and roots of wheat plants between the control and treatment conditions. For G and H, the circle size indicates the number of DEGs, and the rich factor indicates the enrichment degree of the KEGG pathways involving DEGs

In this study, transcriptome sequencing generated more than 1,145,14 million raw reads (Table 1). Among these reads, the GC content of cDNA libraries was approximately 54%. After quality control, 1,134,550,000 clean reads were obtained. The Q_20_ value was more than 98%, and the Q_30_ value was more than 94%. Among them, more than 90% clean reads were mapped to the wheat genome (Table 1). Principal component analysis showed that there were significant differences in the expression patterns between different Fe treatments and different wheat tissues (Fig. 8A). The correlation between gene expression levels in samples is an important indicator for testing the reliability of experiments. In this study, under the same Fe treatment, the *Pearson* correlation coefficient between each pair of biological replications was generally higher than 0.90, which indicated that the similarities between samples regarding transcriptome sequencing were very high (Figure S1). A total of 2,861 genes were identified to be differentially expressed (FDR < 0.05, fold change ≥ 2) between Fe deficiency and Fe sufficiency conditions. There were a total of 137 differentially expressed genes (DEGs) simultaneously identified in both shoots and roots (Fig. 8B). In detail, the number of DEGs (378) in the shoots was relatively smaller than that in the roots under Fe-deficient stress; among them, 313 genes were upregulated, and 65 genes were downregulated (Fig. 8B, C). Of the DEGs (2,619) in the roots under low Fe stress 1,589 were up-regulated and 1,030 down-regulated (Fig. 8B, D). Furthermore, genomic distribution preference analysis of DEGs showed that in the shoots, 128 DEGs were located on the A subgenome, while 126 DEGs were located on the B subgenome, and 124 DEGs were located on the D subgenome (Figure S2). On average, there were 18 DEGs distributed on each chromosome in the shoots. In the roots, a total of 991 DEGs were located on the A subgenome, while 788 DEGs were located on the B subgenome, and 840 DEGs were located on the D subgenome, with an average of 124 DEGs distributed on each chromosome.

**Table 1:** Statistics of wheat transcriptome sequencing data under low Fe stress

Sample	Raw reads	Clean reads	Mapped Reads	Error rate(%)	Q20(%)	Q30(%)	GC content(%)
HIR1	103,367,072	102,485,512	93,941,511 (91.66%)	0.0244	98.24	94.79	55.13
HIR2	92,962,154	91,950,192	82,671,080 (89.91%)	0.0246	98.16	94.60	54.20
HIR3	93,650,802	92,663,246	83,454,929 (90.06%)	0.0246	98.17	94.63	54.67
HIS1	81,831,040	81,022,372	75,929,551 (93.71%)	0.0249	98.03	94.26	55.55
HIS2	97,354,592	96,467,486	90,270,260 (93.58%)	0.0244	98.21	94.75	56.63
HIS3	109,751,026	108,750,122	102,330,323 (94.10%)	0.0244	98.24	94.80	55.66
LIR1	88,487,912	87,710,632	81,340,207 (92.74%)	0.0247	98.12	94.44	55.03
LIR2	84,443,470	83,702,758	76,906,608 (91.88%)	0.0248	98.08	94.38	54.53
LIR3	108,537,716	107,600,262	100,077,545 (93.01%)	0.0245	98.19	94.64	55.20
LIS1	85,080,798	84,380,564	79,656,284 (94.40%)	0.0241	98.33	95.06	57.10
LIS2	106,018,904	105,038,702	98,685,159 (93.95%)	0.0245	98.19	94.67	55.62
LIS3	93,658,786	92,779,306	87,098,185 (93.88%)	0.0247	98.10	94.43	55.92

*HIR* Hight Iron Root, *HIS* Hight Iron Shoot, *LIR* Low Iron Root, *LIS* Low Iron Shoot. 1, 2, and 3 respectively represent different biological repetitions. Error rate (%) is the average error rate of sequencing bases corresponding to the quality control data. Q20% is the proportion of the nucleotide quality value larger than 20; Q30% is the proportion of the nucleotide quality value larger than 30; GC content (%) is proportion of guanidine and cytosine nucleotides among the total nucleotides

To obtain functional information for the DEGs, a GO annotation analysis was performed. The GO items were classified into three types: biological process (BP), cell component (CC), and molecular function (MF). GO entries with a *p* value < 0.001 were considered to be significantly enriched. In both shoots and roots under low Fe, BP was mainly enriched in the metabolic process and cellular process (Fig. 8E), while CC was mainly enriched in the cell part and membrane part terms (Fig. 8E); in the MF category, binding and catalytic activity were the two most abundant GO terms (Fig. 8E). KEGG annotation divided the pathways in which DEGs participated into five categories: cellular processes, environmental information processing, genetic information processing, metabolism, and organismal systems. Most DEGs in the cellular processes category were annotated to the endocytosis pathway, and most DEGs enriched in the environmental information processing category were annotated to the MAPK signaling pathway–plant pathway. In contrast, most DEGs involved in the genetic information processing category were annotated to protein processing in endoplasmic reticulum pathway, whereas most of the DEGs involved in the metabolism category were annotated to the phenylpropanoid biosynthesis pathway, and most DEGs belonging to organic systems were annotated to the plant-pathogen interaction pathway (Fig. 8F).

To further deepen our understanding of the functionality of these DEGs, we conducted a more detailed GO enrichment analysis (Fig. 8G). GO terms with a *p* value < 0.001 were considered to be significantly enriched. We found that most DEGs were related to plant metabolism and biosynthesis processes, such as the tricarboxylic acid biosynthetic process, nicotianamine synthase activity, the nicotianamine metabolic process, the nicotianamine biosynthetic process, nicotianamine aminotransferase activity, and the L − methionine biosynthetic process. These results indicated that Fe chelator biosynthesis and Fe transport were implicated in maintaining Fe homeostasis. Significantly enriched GO terms related to ion transport and ion homeostasis, which included Fe transport and intracellular Fe sequestration In addition, the GO terms related to photosynthesis, such as photosynthesis and light harvesting in photosystem I, were also highly accumulated. These results emphasized the importance of membrane and/or membrane-localized metal ion transporters and regulatory and metabolic proteins under Fe deficiency.

The KEGG database was used to further determine the pathways involved in the responses of wheat to low-Fe stress (Fig. 8H). Previous studies have shown that the accumulation of amino acids is believed to have a beneficial effect on the adaptation of plants to stresses [16–18]. Most of the DEGs were identified to be involved in amino acid metabolism pathways, such as alanine, aspartate and glutamate metabolism, tyrosine metabolism, cysteine metabolism, methionine metabolism, phenylalanine metabolism, and other KEGG pathways. The DEGs were also implicated in pathways related to photosynthesis, such as photosynthesis-antenna proteins and carotenoid biosynthesis. In addition, phenylpropanoid biosynthesis, MAPK signalling pathway-plant, and other KEGG pathways were also significantly enriched (Fig. 8H).

### Transcriptional profiling of Fe transport-related genes under low Fe

GO enrichment indicated that the biosynthesis of Fe chelators and Fe transport played an important role in the responses of wheat plants to low Fe stress. Among the DEGs, genes related to Fe homeostasis are the key genes for the adaptive responses of wheat plants to low Fe. Therefore, we analysed the expression of genes related to Fe uptake and transport under Fe deficiency stress. Figure S3 shows a molecular model of genes involved in Fe absorption and transport in plant roots (Figure S3A), chloroplasts, mitochondria, and vacuoles (Figure S3B). Transcriptional profiling results showed that most of the key genes, such as Fe^3+^ chelate reductase (*FRO*), iron-regulated transporter (*IRT*), natural resistance-associated macrophage protein (*NRAMP*), and yellow stripe-like (*YSL*), involved in efficient Fe uptake belonging toStrategy I, were significantly upregulated under Fe deficiency. Among them, *TaFRO2-2A* (Figure S4A) and *TaIRT1a-4A* (Figure S4E), *TaNRAMP2-4A* (Figure S4B), and *TaYSL15a-6D* (Figure S4C) were upregulated by three, six, five, and 70-fold, respectively.

In Strategy II, the expression levels of genes involved in plant Fe siderophore biosynthesis and Fe uptake were significantly upregulated. The process of biosynthesis and secretion of plant Fe siderophores is very complicated. It requires the participation of 13 enzymes, four of which are key enzymes: methionine synthetase (SAM), nicotinamide synthase (NAS), nicotinamide aminotransferase (NAAT), and deoxygenate synthase (DMAS). Differential gene expression showed that these four key enzyme genes were significantly induced under low Fe stress. Among them, *TaSAM3-6B* (Figure S4D), *TaNAS1c-6D* (Figure S4I), *TaNAAT1b-1B* (Figure S4G), and *TaDMAS1-4A* (Figure S4F), showing the most significant changes in expression levels, were upregulated by 3, 3105, and 2718, and 24-fold, respectively. In Strategy II, the genes encoding transporter of mugineic acid (TOM) (Figure S4J) and multidrug and toxin efflux family (*MATE*) (Figure S4K) members were significantly upregulated under low Fe stress. Among them, the expression of *TaTOM-2B* and *TaMATE-4A* was changed the most, upregulated by two and 20 folds, respectively. Oligopeptide transporter (OPT) is a polypeptide transporter located on the plasma membrane of plant epidermal cells. Transcriptomic analysis showed that the *TaOPT* homologues were significantly induced by low Fe, and the expression of *TaOPT3-5B* increased by 44 fold under low Fe (Figure S4M).

The vacuolar iron transporter (*VIT*)-mediated vacuolar loading of Fe into seed embryos not only promotes seed development but is also crucial for seed germination. *NRAMP*, located on the vacuole membrane, mediates the transport of Fe from the vacuole of the seed embryo to the cytoplasm and participates in the supply of Fe during seed germination. The expression levels of most *VIT* genes were significantly downregulated under low Fe stress, particularly *TaVIT2-5B*, which was downregulated threefold (Figure S4R), and might play a key role in promoting seed development. *NRAMP3/4* was significantly induced by low Fe stress; among these DEGs, *TaNRAMP3-7D*, which was upregulated threefold, might play an important role in the transport of Fe from the seed embryo vacuole to the cytoplasm (Figure S4B). Mitochondrial m-type thioredoxin in chloroplasts (*ATM*) is mainly involved in the process of exporting Fe-S to mitochondria. *TaATM-5A* had the highest expression abundance and might play an important role in maintaining mitochondrial Fe balance (Figure S4H). Permease in chloroplasts (*PIC*) was the first protein identified to participate in chloroplast Fe transport, and its expression was not found to be regulated by Fe in other crop species. *PIC* was significantly induced by low Fe in the shoots of wheat plants, and *TaPIC-1A* had the highest expression abundance (Figure S4L). The iron efflux transporter ferroportin (*FPN*), located in the chloroplast, can regulate intracellular Fe content by participating in the transport of NA or the Fe-NA complex into the chloroplast. *FPN* was significantly induced by low Fe in the roots, and *TaFPN1-7A* was significantly upregulated by 60-fold (Figure S4P). Nonintrinsic ABC protein (*NAP*) located on the inner chloroplast membrane encodes a non-plasma membrane-localized nucleotide-binding domain subunit of the ABC transporter. *NAP* was significantly induced by low Fe in the shoots, and *TaNAP-3A* was significantly upregulated (Figure S4Q). Another contributor to promoting the transfer of Fe into the chloroplast is mitoferrin-like proteins, which contains chloroplast transit peptides and is mainly located in rosette leaves. The expression levels of most mitoferrin or mitoferrin-like genes significantly decreased under low Fe stress (Figure S4N, O).

### Transcriptional profiling of photosynthesis-related genes under low Fe

The GO enrichment analysis showed that GO terms related to photosynthesis, such as photosynthesis and light harvesting in photosystem I, were significantly enriched (Fig. 8G). Moreover, the KEGG enrichment analysis also showed that photosynthesis-antenna proteins and carotenoid biosynthesis were significantly enriched under low Fe stress (Fig. 8H). Many genes related to photosynthesis, such as photosynthetic antenna proteins and key genes in the biosynthesis of carotenoids were identified (Figure S5). Through genomic annotation, a total of 33 genes encoding photosynthesis-antenna proteins (Figure S5A) and 39 genes encoding carotenoid biosynthesis (Figure S5B) were retrieved in the wheat genome. The results showed that the expression levels of most genes involved in the photosynthetic antenna protein pathway and carotenoid biosynthetic pathway were significantly induced by low Fe (Figure S5A, B). The genes encoding chlorophyll a oxygenase (*CAO*), chlorophyll b reductase (*CBR*), chlorophyll synthase (*CS*), and chlorophyllase play an important role in the conversion between chlorophyll a and chlorophyll b. Among the DEGs involved in photosynthesis, we found that the genes encoding these enzymes showed an increased expression pattern under low Fe stress (Figure S5C). In terms of plant carotenoid biosynthesis, geranylgeranyl diphosphate (GGPP) generates the first carotenoid substance phytoene. After dehydrogenation, cyclization, hydroxylation, epoxidation, etc., phytoene is transformed into other carotenoids. The expression of key genes implicated in carotenoid biosynthesis, such as phytoene synthase (*PSY*), β-carotene hydroxylase (*β-OHase*), zeaxanthin epoxidase (*ZEP*), 9-cis-epoxycarotenoid dioxygenase (*NCED*), *LUT5*, and violaxanthin de-epoxidase (*VDE*), was significantly reduced under low Fe stress (Figure S5D). Moreover, the expression of chlorophyll a/b binding proteins (Figure S5E), photosynthesis II reaction proteins (Figure S5F), RuBisCo subunit binding proteins (Figure S3G), Mg^2+^ chelatase (Figure S5H), and ribulose bisphosphate carboxylase/oxygenase activase (Figure S5I) wassignificantly downregulated under low Fe stress.

### Transcriptional profiling of other ion transporters under low Fe

**Fig. 9:**
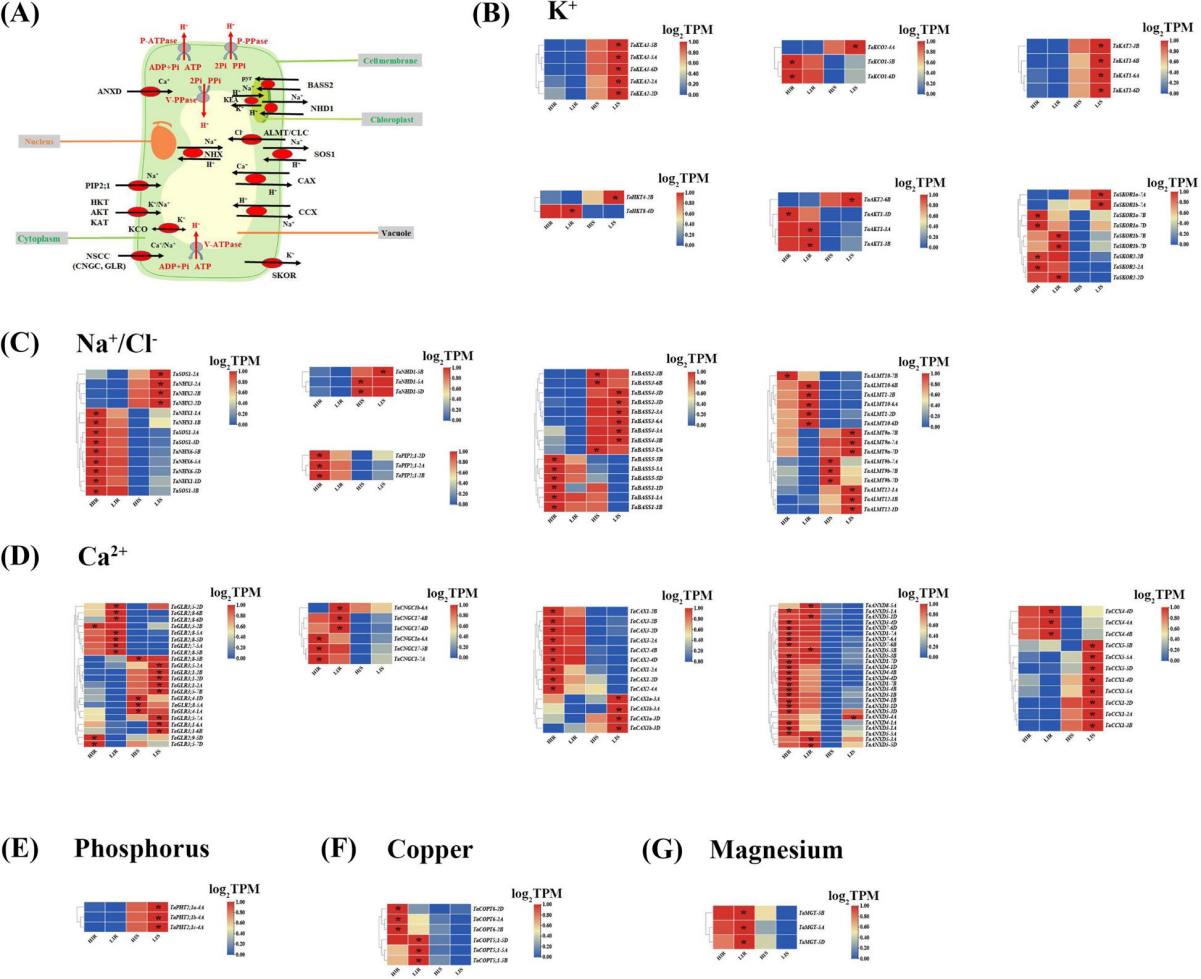
Differential expression profiling of other ion transport genes in wheat plants under low Fe stress. (**A**) Molecular model of genes responsible for the transport of other cations. Differential expression profiling of the genes involved in the transport of potassium (K^+^) (**B**), sodium (Na^+^)/chlorion (Cl^−^) (**C**), calcium (Ca^2+^) (**D**), phosphorus (Pi) (**E**), copper (Cu^2+^) (**F**), and magnesium (Mg^2+^) (**G**) ions. The heat maps show the gene expression levels indicated by TPM values. Differentially expressed genes that show higher expression levels under the control (high Fe: 50 μM) and treatment (low Fe: 2 μM) are indicated by asterisks

Among the numerous DEGs, genes related to ion homeostasis are the key genes involved in wheat resistance to Fe deficiency stress. Figure 9A shows a molecular model of the key genes responsible for regulating K^+^, Na^+^/Cl^−^, and Ca^2+^ transport. Most of the K^+^ transporter genes, including the K^+^ efflux transporter gene *KEA* located on the chloroplast, the vacuolar K^+^ inflow transporter gene *KCO*, the plasma membrane-located K^+^ inflow transporter gene *AKT/KAT* and *HKT*, and the externally rectifying K^+^ channel *SKOR*, were upregulated under low Fe stress (Fig. 9B). The gene expression levels of vacuolar Na^+^/H^+^ antiporters (NHXs), particularly *NHX2,* which is involved in the vacuolar Na^+^ compartment, and *SOS1/NHX7,* which regulates cellular Na^+^ exclusion, were significantly upregulated (Fig. 9C). *NHD* regulates the efflux of Na^+^, and the expression of *TaNHD1-5B* was significantly up-regulated twofold, which might help reduce chloroplast damage caused by excessive Na^+^ (Fig. 9C). The chloroplast-localized bile acid:Na^+^ cotransporter (*BASS*) regulates Na^+^ influx, and its expression level was significantly inhibited by low Fe stress, particularly *TaBASS1D-1D* (Fig. 9C). In addition, low Fe stress also induced the expression of most *ALMT* genes, which were related to the transport of Cl^−^ in vacuoles; the expression of *TaALMT12-1B* was significantly up-regulated by two folds under low Fe stress (Fig. 9C). Subsequently, the expression of genes related to Na^+^/Ca^2+^ transport, including *CCX*, *CAX*, *ANXD*, *GLR*, and *CNGC,* was investigated. The expression levels of most genes changed significantly under low Fe stress (Fig. 9D). For example, under low Fe stress, *TaCCX* expression increased, *TaCAX* expression was decreased, and *TaANXD* expression increased (Fig. 9D). This might be an important reason for reducing cytoplasmic Na^+^ and increasing cytoplasmic Ca^2+^ concentrations. The expression of most of the plasma membrane-localized *NSCC* genes, including *GLR* and *CNGC*, were up-regulated under low Fe stress (Fig. 9D). In addition, under low Fe stress, the expression levels of *PHT2;1* (Fig. 9E) and *COPT* (Fig. 9F), which are involved in the root uptake and transport of Pi and Cu^2^ + , were significantly downregulated, while the genes involved in Mg^2+^ uptake and transport were significantly induced by low Fe stress (Fig. 9G). The above results all indicated that the maintenance of ion homeostasis in cells was important in the plant responses to low Fe stress.

### Transcriptional profiling of cell cycle-related genes and ROS metabolism-related genes under low Fe

To study whether the inhibitory effect of Fe deficiency on root growth was related to the degree of cell division and differentiation, we utilized transcriptome data to analyse the expression of cell cycle-related genes (Figure S6A). Cyclin and cyclin-dependent kinases, which are cell cycle control proteins, play key roles in the process of mitosis [19]. *LRP* (lateral root primordia), specifically expressed in adventitious roots and lateral root primordia, is a transcription factor that regulates root elongation [19]. Under low Fe stress, the expression abundances of these genes were significantly reduced (Figure S6A). This result indicated that the shortening of roots under low Fe stress might be attributed to the decrease in the division frequency of cells in the meristematic zone and the reduced cell elongation in the elongation zone.

According to the results in Fig. 5 and Fig. 6, we further analysed the expression of key enzyme genes in the ROS metabolic pathway (Figure S6B, C). Respiratory burst oxidase homologous genes (*RBOH*) encoding NADPH oxidase play key roles in the production of ROS [20]. Under low Fe stress, a total of 11 RBOH DEGs were significantly upregulated in the shoots or roots (Figure S6B). The general expression of *SOD* and *CAT*, as well as genes in the shoots and roots, was increased under low Fe stress, which might be necessary for ROS scavenging under low Fe stress (Figure S6C).

### Transcriptomics-assisted gene coexpression network analysis

In allohexaploid wheat, multiple copies of genes are ubiquitous within a gene family. Therefore, the identification of core genes is an important prerequisite for understanding the molecular mechanisms underlying the regulation of important agronomic traits. Systematic analysis of the transcriptional responses of genes related to Fe uptake and transport under low Fe stress will help us to fully understand the adaptative responses of wheat plants to Fe deficiency. To identify the core members of genes related to Fe uptake and transport, we constructed gene coexpression networks (Figure S7A). The results showed that some Fe^2+^ absorption-related and Fe^2+^ transport-related genes, including *TaIRT1b-4A*, and plant siderophores-related genes, such as *TaNAS2-6D*, *TaNAS1a-6A*, *TaNAS1a-6B* and *TaNAAT1b-1D*, were identified as core target genes (Figure S7A) that might play key roles in wheat responses to low Fe stress.

To study the roles of the five key genes involved in wheat tolerance to low Fe stress, we performed a population screening of 386 wheat varieties to identify those with different levels of tolerance to low Fe stress. The results indicated that the root lengths of wheat plants ranged from 10.6 to 50.4 with a median of 30.5. Notably, these values were normally distributed with a CV of 30.95% (Figure S8A). The chlorophyll content (represented by SPAD values) ranged from 1.0 to 50.4 with a median of 27.9. Notably, these values were normally distributed with a CV of 40.95% (Figure S8A), suggesting that a wide range in natural variation in low Fe tolerance exists among the different wheat genotypes. Finally, we identified two wheat cultivars with different levels of tolerance to low Fe: the low-Fe-sensitive cultivar Zhengmai 1860 and the low-Fe-tolerant cultivar Zhoumai32 (Figure S8B). The results showed that, regardless of Fe supply, the leaves of Zhoumai32 were always green. However, the leaves of Zhengmai1860 were pale yellow or whitish under low Fe stress, its young leaves were chlorotic, and yellowing was evident between leaf veins (Figure S8B). Next, we quantitatively analysed the expression levels of the five core genes initially identified in these two varieties (Figure S8C). The results showed that none of the five genes were significantly different in the shoots but were significantly induced by low Fe stress in the roots. In addition, the expression levels of the five core genes were upregulated at a higher fold in the low-Fe-tolerant variety Zhoumai32 than in the low-Fe-sensitive variety Zhengmai 1860, which highlighted that *TaIRT1b-4A*, *TaNAS2-6D*, *TaNAS1a-6A*, *TaNAS1a-6B* and *TaNAAT1b-1D* might play key roles in wheat tolerance to low Fe stress.

## Discussion

Low Fe stress severely inhibits plant growth, seed yield, and crop quality worldwide. Allohexaploid wheat is a major food crop that is highly sensitive to Fe deficiency. Dissecting the physiological and molecular mechanisms underlying wheat tolerance to low Fe stress will provide a theoretical basis for breeding new wheat varieties with high Fe use efficiency.

### Morpho-physiological responses of wheat plants to low Fe stress

In higher plants, Fe deficiency is characterized by chlorosis between the veins of young leaves, whereas mature leaves remain green due to Fe immobility [21]. In this study, the wheat seedlings studied showed obvious damage, including yellowing of the leaves (Fig. 1) and inhibition of root growth under low Fe stress (Figs. 1, 3). Although Fe is not a component of chlorophyll, it is indispensable in the photosynthetic process [22]. The degree of leaf xanthosis caused by low Fe in crops is an effective indicator for judging whether crops lack Fe [23, 24]. As a functional indicator of photosynthetic pigment and light response [25], the ratio of the concentrations of chlorophyll a to chlorophyll b under low Fe stress was significantly lower than under the control condition (Fig. 2D). This result indicated that low Fe stress had a more obvious inhibitory effect on chlorophyll a catabolism. In addition, the concentrations of both total chlorophyll and carotenoids were decreased under low Fe stress (Fig. 2D, F), which also suggested that photosynthetic pigments were severely degraded under low Fe stress [26]. Severe nutrient deficiency affects the cell and subcellular morphology of plant species [27, 28]. Previous studies in rice [27] and wheat [28] found that the reduced number or destruction of chloroplasts inhibited the photosynthetic rate. In this study, low Fe stress caused chloroplast separation and chlorophyll degradation, which might further lead to photosynthesis inhibition (Fig. 2D-F, Fig. 3A). Differential gene expression profiling showed that the photosynthesis-related KEGG pathway was highly enriched (Fig. 8H), and the genes related to the chlorophyll biosynthesis pathway were significantly downregulated under low Fe stress (Figure S5). The root system is a major organ for wheat plants to absorb essential mineral elements for growth and development. Programmed cell death (PCD) is responsible for the selective elimination of damaged or unwanted cells and organs to maintain cellular homeostasis under normal or stress conditions [29]. Since root growth is related to the rate of cell division and elongation, ultrastructure analysis of wheat roots showed that under low Fe stress, cell division was reduced, resulting in a reduction in the elongation of meristem cells, which was consistent with the observed reduction in the growth of whole roots (Fig. 2B, C).

Some previous studies showed that under low Fe, the contents of ROS and osmotic adjustment substances changed significantly [30]. When adversity stress forces ROS production in plant cells, it will cause damage to macromolecular substances and other components in the cell, hindering the normal metabolism and growth of plants and even causing death [31]. Our results showed that under low Fe conditions, the concentrations of OFR and H_2_O_2_ increased significantly (Fig. 5B, C), which initiated membrane lipid peroxidation and damaged the structure of the cell membrane system. However, the root ion leakage rate (Fig. 2G) and MDA content (Fig. 5D) under Fe deficiency were not different from those under control conditions, indicating that the accumulation of ROS did not cause great damage to cell membranes in the wheat roots. Osmotic regulators, such as Pro and MDA, were highly accumulated in the cells (Fig. 5A, D), thereby regulating osmotic potential and further maintaining normal metabolism in cells.

### Transcriptomics-assisted analysis of ionomic responses of wheat plants to low Fe stress

Fe homeostasis in plants is jointly modulated by a variety of Fe ligands, transporters, and regulatory factors. Usually, Fe is combined with ligands for transportation or storage to reduce the risk of toxic ROS produced by free Fe [32]. NA and deoxysarcosine (DMA) are key Fe chelating agents and are implicated in efficient Fe acquisition and transport. NA has long-distance and cellular Fe transport functions in higher plants [33–35]. DMA is mainly involved in long-distance Fe transportation in the xylem and phloem [36, 37]. In the present study, we found that the GO terms related to the biosynthesis of both NA and DAM were significantly enriched in our transcriptome data (Fig. 8G). In addition, the expression of key enzymes in the NA and DAM synthesis pathways, such as *SAM*, *NAS*, *NAAT*, and *DMAS*, was significantly upregulated (Figure S4D, I, G, F), which was consistent with previous results reported in wheat [38–40] and rice [33–35]. Previous studies have shown that the expression levels of genes related to Fe uptake and transport in mitochondria [41, 42], chloroplasts [43, 44], and vacuoles [45] are significantly upregulated under Fe-deficiency stress. This conclusion was consistent with our transcriptomic results (Fig. S3, Fig. S4). The increased expression of these genes in young leaves under low Fe stress might contribute to the unloading of more Fe into mesophyll cells to meet the normal absorption and utilization of Fe by young leaves. However, when the Fe content in the cell exceeds its limit, the plant can transport Fe^2+^ chelates into the organelles, such as vacuoles, to store Fe, thus avoiding cell Fe toxicity. Studies have shown that when *AtVTL1*, *AtVTL2*, or *AtVTL5* is expressed under the 35S promoter in the background of two mutants of *nramp3*/*nramp4* or *vit1-1* [46], *AtVTL1*, *AtVTL2* or *AtVTL5* can restore root growth. In yeast, the VIT homologous Ca^2+^-Sensitive-Cross-Complementer (CCC1) protein is a vacuolar Fe transporter that transports Fe to the vacuole under conditions of excess Fe [45]. The pastatin promoter was used for overexpression in cassava, and the expression of the *AtVTL1* gene was positively correlated with the increase in Fe concentrations in storage roots. This result shows that the *VIT* gene is involved in mediating vacuolar Fe retention [47]. The above results all indicate that the dynamic balance of Fe in cells is essential for the normal growth and development of plants.

### Transcriptomics-assisted molecular responses of wheat plants to Fe deficiency

Abiotic stress can negatively affect plant growth and development, resulting in yield loss. It is estimated that more than 90% of the world's agricultural land is affected by abiotic stressors. However, different crops have different responses to low Fe stress, such as Arabidopsis [48], wheat [49, 50] and maize [51]. In Arabidopsis [48], the transcriptional levels of IRT1, FRO2 and bHLH100 were highly increased (57-, 58-, and 357-fold, respectively), suggesting that these genes play important roles in Arabidopsis responses to low-Fe stress. Previous studies have shown that in wheat [49, 50], the significantly enriched GO entries were mainly related to translation, cellular protein metabolism, and protein metabolism and transport under low Fe, which was consistent with the transcriptomic results in this study. In addition, the expression levels of *OsVIT2*, *OsFER2*, *OsFER2* and other genes in rice were significantly upregulated under low Fe stress, thereby allowing the plants to respond to low Fe stress, which is consistent with the results of this study. Fe deficiency can significantly inhibit maize growth, resulting in decreased chlorophyll and active Fe content [21, 51]. In addition, Fe deficiency stress significantly induced genes related to DMA biosynthesis, secretion, and Fe(III)-DMA transport, resulting in the synthesis and secretion of more DMA and the development of more lateral roots[51]. In conclusion, the theoretical basis of plant responses to low Fe stress is similar.

## Conclusions

Fe nutrients play important roles not only in plant physiological functions but also in maintaining various cell processes. In the past few decades, increasing progress has been made in understanding how plants adapt to low Fe availability in soils. However, observing the dynamics of Fe nutrient status in plants is still a significant challenge. The genes responsible for Fe absorption, translocation from roots to shoots, storage in cells and even their regulation at the transcriptional and posttranscriptional levels are being characterized in crop species. However, further studies are still needed to reveal the further connection and integration between Fe-deficient signalling pathways and developmental and physiological networks. Our study analysed the morphological and physiological changes in the shoots and roots under low Fe stress and utilized the transcriptome to analyze the expression profiles of genes related to Fe uptake and transport, photosynthesis, and other ion transporter-related genes. Finally, key genes affecting the plant responses to low Fe stress were identified. In summary, these results might provide excellent genetic resources for the regulatory mechanisms underlying adaptability to low Fe stress and high Fe use efficiency in wheat.

## Materials and methods

### Plant material and low Fe stress treatment

The bread wheat (*Triticum aestivum* L.) cultivar, Zhengmai 1860 that is bred by Henan Academy of Agricultural Sciences (Zhengzhou, 450,001, China), was used in this study. The seeds of Zhengmai 1860 on wet gauze were germinated for 7 days, and then the seedlings with uniform sizes were selected for hydroponic culture for 10 days in black plastic boxes containing 10 L of Hoagland nutrient solution (pH 5.8). Half of the seedlings were grown in a nutrient solution containing sufficient Fe, and half were grown in a nutrient solution containing low Fe stress. The regimes of hydroponic solutions were as follows: 1.0 mM KH_2_PO_4_, 5.0 mM KNO_3_, 5.0 mM Ca(NO_3_)_2_·4H_2_O, 2.0 mM MgSO_4_·7H_2_O, 9.0 μM MnCl_2_·4H_2_O, 0.80 μM ZnSO_4_·7H_2_O, 0.30 μM CuSO_4_·5H_2_O, 0.10 μM Na_2_MoO_4_·2H_2_O, and 46 μM H_3_BO_3_ with different Fe(III)-EDTA concentrations (Fe-sufficient condition: 50 μM; low Fe condition: 2 μM). The growth condition was as follows: light intensity of 200 μmol m^−2^ s^−1^, room temperature of 25 °C daytime/22 °C night, light period of 16 h photoperiod/8 h dark, and relative humidity of 70%. The nutrient solution was refreshed every 5 days, and its pH was constantly adjusted to 5.8 with NaOH.

A total of 386 wheat accessions were collected for screening of wheat genotypes with different low Fe tolerance (Table S1). The Fe treatments and growth conditions of these wheat seeds were as described above. Primary root length and photosynthetic pigment content of wheat plants were measured after 10 days of low Fe treatment. Gene-specificity primers used for the assays of relative expression of target genes are shown in Table S2.

### Determination of physiological indexes

After the 10-d treatment, the shoots and roots of were taken from at least five uniform wheat seedlings, and their fresh weights were measured. Then, after oven-dried at 65 °C to constant weight, the dry biomasses of wheat plants were weighed, and then the root-to-shoot ratio and relative shoot water content were calculated. According to a previous study [52], 0.2 g of the leaf pieces were weighed to determine total chlorophyll content and placed in a test tube containing 5 mL of 80% acetone solution and were soaked in dark at 25 °C for 24 h. 80% acetone solution was used as the blank, and a DU®640 UV–Vis spectrophotometer (Beckman) was used to measure the absorbance at wavelengths of 470 nm, 645 nm, and 663 nm, respectively.

Fresh root samples were incubated and shaked (100 r/min) in 5 mL 0.4 M mannitol at 25 °C for 4 h, and then we used the conductivity meter (DDSJ-318, Shanghai, China) to measure the solution conductivity as the initial leakage rate. Then, the samples in a water bath were put at 85 °C for 20 min, the solution leakage rate was measured and recorded as the total leakage rate. The ion leakage rate was calculated as the ratio of the initial leakage rate to the total leakage rate [53].

A root scanner (Microtek Scan Maker I800, WinRHIZO Pro) was used to measure total root length, maximal root length, root surface area, root volume, root tip number, lateral root length, and average root diameter of wheat plants. Root activity of wheat plants under different treatments was determined by the tetrazolium (TTC) reduction method. The Comin biological kit (http://www.cominbio.com) was used to determine the content of proline, superoxide anion (OFR), hydrogen peroxide (H_2_O_2_), and malondialdehyde (MDA) in the shoots and roots of wheat plants under different Fe treatments. Five biological replicates were prepared for each treatment.

### Microscopy analysis

After exposed to 10-d low Fe stress, the roots of wheat plants were taken and placed in a glass container filled with ddH_2_O. The morphology of root hairs was observed with a stereoscopic microscope (Leica M125C).

Paraffin sections were used to investigate cell sizes in wheat root tips. First, fresh wheat root tips were added into FAA fixative (formaldehyde: glacial acetic acid: 70% ethanol = 1:1:18). It was then embedded after dehydration, transparency, and wax dipping. The slice thickness was approximately 8–10 μm, and a typical section was selected for microscopy (Leica M125C) [54].

The shoots of wheat plants under different treatments were fixed in fresh 4% glutaraldehyde fixative for 12 h. Then, the samples were rinsed with 0.1 mol/L phosphate-buffered solution (PBS) of pH 6.8, and then the rinsed samples were put into 1% osmium acid solution for fixation at 4℃ for 1 h, and then were dehydrated with 100% ethanol. The resultant samples were subjected to infiltration, resin embedding and polymerization, and be stored in a desiccator. The samples were cut into ultrathin sections of 50–70 nm, double-stained with uranyl acetate-lead citrate, observed, and photographed by a transmission electron microscope (H-7650; Hitachi, Tokyo, Japan) [55, 56].

### Detection of ROS in wheat plants

A common consequence of abiotic stress in plants is ROS production, and the accumulation of ROS can be estimated from the histochemical staining intensity of total H_2_O_2_ and O_2_^¯^ [57]. 3′3’-Diaminobenzidine (DAB) can react with H_2_O_2_ in the presence of peroxidase to form a dark brown polymer product, thereby detecting the H_2_O_2_ content in plant tissues [58]. Nitro blue tetrazole (NBT) can react with oxygen-free radical O_2_^¯^ to produce dark blue insoluble formazan, which can be used to detect the O_2_^¯^ content in plant tissues [59].

### Ionomic analysis

Uniform wheat seedlings after 7-d seed germination were hydroponically grown under sufficient Fe and low Fe conditions for 10 days until sampling. Over-dried shoot and root tissues were subsequently transferred to an HNO_3_/HClO_4_ mixture (4:1, v/v) at 200℃ until completed digestion. The diluted supernatant was determined to quantify the concentrations of mineral elements using an inductively coupled plasma mass spectrometry (ICP-MS; NexIONTM 350X, PerkinElmer). Each sample contained five independent biological replicates for the ionomic analyses under low Fe stress.

### Transcriptomic analysis

To understand the molecular basis of Fe tolerance in wheat seedlings under low Fe stress, we constructed RNA-seq libraries of the wheat shoots and roots [60, 61]. Under low Fe stress, the 7-d-old uniform seedlings after seed germination were hydroponically grown under Fe-sufficient and low-Fe solution for 10 days until sampling. The shoots and roots of the afore-mentioned wheat plants were harvested, and three independent biological replicates were used for each treatment. Pre-chilled Trizol (Takara Bio Inc, Kusatsu, Shiga, Japan) was used to isolate total RNA. The RIN (RNA integrity number) values (> 8.0) of these samples were assessed using an Agilent 2100 Bioanalyzer (Santa Clara, CA, USA). Samples of RNA with the RIN values > 8.0 were obtained to construct strand-specific cDNA libraries, which were further used for paired-end sequencing (read length = 150 bp) on a lane of an Illumina Hiseq 4000 platform. The sequenced data were assembled against the reference genome sequences of the bread wheat variety Chinese Spring (IWGSC RefSeq v1.1 assembly,. and Kyoto Encyclopedia of Genes and Genomes (KEGG) and Gene Ontology (GO) were used for functional enrichment analyis of DEGs [62–66]. The values of Transcripts Per Kilobase of exon model per Million mapped reads (TPM) were normalized to quantify gene expression levels, and both false discovery rate and *P* values < 0.05 were used to identify the DEGs [67]. PANTHER (http://www.pantherdb.org/data/) [68] and KEGG (http://www.kegg.jp/) [69] were used to perform GO and pathway enrichment analysis of the DEGs, respectively. Heat maps showing differential gene expression were delineated using a Multiexperiment Viewer (http://www.tm4.org/mev.html) [70].

### Reverse transcription quantitative PCR assays

Reverse transcription quantitative PCR (RT-qPCR) assays were performed to validate the accuracy of transcriptome sequencing data. After removing genomic DNA from RNA samples with RNase-free DNase I, total RNA was used as RT templates for cDNA synthesis using the PrimeScript™ RT Reagent Kit Eraser (Perfect Real Time; TaKaRa, Shiga, Japan). The RT-qPCR assays were performed to detect relative gene expression using SYBR®Premix Ex Taq™ II (TliRNaseH Plus) (TaKaRa, Shiga, Japan) using a Bio-Rad C1000 Touch Thermal Cycler of CFX96™ Real-time PCR detection system.

The RT-qPCR program was as follows: 95ºC for 3 min, 40 cycles of 95ºC for 10 s, and 60ºC for 30 s. The melting curve was plotted as follows to analyse the primer gene-specificity: 95ºC for 15 s, 60ºC for 1 min, and 60-95ºC for 15 s (+ 0.3ºC/cycle). The expression data of target genes were normalized, and the relative expression levels were calculated according to the 2^−ΔΔC^_*T*_ method [71]. Each sample contained three independent biological replicates.

### Gene coexpression network analysis

Gene coexpression networks were constructed to identify gene interactions and locate core genes that connect most neighboring genes involved in the responses of wheat plants to low Fe stress. For each pair of genes, the threshold of the *Pearson* correlation value was set according to the default settings (http://plantgrn.noble.org/DeGNServer/Analysis.jsp), and then gene coexpression networks were constructed by CYTOSCAPE 3.2.1 (Donnelly Centre for Cellular and Biomolecular Research, University of Toronto, Toronto, Canada) (http://www.cytoscape.org/) [72].

### Statistical analysis

Significant differences (*, *P* < 0.05; **, *P* < 0.01; ***, *P* < 0.001) were determined by unpaired two-tailed Student’s *t*-test using Statistical Productions and Service Solutions 17.0 (SPSS, Chicago, IL, USA).

## Supplementary Information

**Additional file: Table S1.** Names of wheat accessions used in this study.

**Additionalfile 2: Figure S1.** Correlation analysis of shoot and root samples. HIR:High Iron Root, HIS: High Iron Shoot, LIR: Low Iron Root, LIS: Low Iron Shoot.1, 2, and 3 respectively represent different biological repetitions. **Figure S2.** Genomic distribution ofdifferentially expressed genes (DEGs). Genomic distribution of DEGs in the shoots (A)and roots (B). **Figure S3.** Molecularmodel of Fe absorption and transport related genes. A molecular model of genesinvolved in Fe absorption and transport in plant roots (A), chloroplasts,mitochondria, and vacuoles (B). **FigureS4.** Differential expression profiles of genes related to Fe absorption andtransport in wheat plants under low Fe stress. (A) Fe^3+^ chelatereductase (*FRO*), (B) naturalresistance-associated macrophage protein (*NRAPM*),(C) Yellow stripe-like (*YSL*), (D) methionine synthetase (*SAM*), (E) iron-regulated transporter (*IRT*), (F) Deoxyergate synthase (*DMAS*), (G) Nicotinamide aminotransferase(*NAAT*), (H) Mitochondrial m-typethioredoxin in chloroplast (*ATM*), (I)Nicotinamide synthase (*NAS*), (J)transporter of mugnetic acid (*TOM*),(K) multidrug and toxin efflux family (*MATE*),(L) Permease in chioroplasts (*PIC*),(M) Oligopeptide transporter (*OPT*),(N) mitoferrin, (O) mitoferrin-like, (P) Iron efflux transporter ferroportin (*FPN*), (Q) Nonintrinsic ABC protein (*NAP*), (R) vacuolar iron transporterginseng (*VIT*). For transcriptomesequencing, selected uniform wheat plants after germination, half of which weretransplanted into a nutrient solution with normal Fe concentration forcultivation, and half were transplanted into a nutrient solution with low Fefor cultivation, and samples were taken 10 days later. The heat map shows thegene expression level indicated by the TPM value. Differentially expressedgenes that show higher expression levels under the control (normal Fe: 50 μM)and treatment (low Fe: 2 μM) are indicated by asterisks. **Figure S5.** Differential expression profile ofphotosynthesis-related genes in wheat plants under low Fe stress. Differentialexpression profile of genes related to photosynthesis-antenna proteins (A) andcarotenoid biosynthesis (B). The expression pattern of genes involved in thetransformation of chlorophyll a and chlorophyll b (C) and the synthesis ofcarotenoids under low Fe stress (D). The expression pattern of chlorophyll a/bbinding proteins (E), Photosynthesis II reaction proteins (F), RuBisCo subunitbinding proteins (G), Mg^2+^ chelatase (H), Ribulose bisphosphatecarboxylase/oxygenase activase (I). For transcriptome sequencing, selecteduniform wheat plants after germination, half of which were transplanted into anutrient solution with normal Fe concentration for cultivation, and half weretransplanted into a nutrient solution with low Fe for cultivation, and sampleswere taken 10 days later. The heat map shows the gene expression levelindicated by the TPM value. Differentially expressed genes that show higherexpression levels under he control (normal Fe: 50 μM) and treatment (low Fe: 2μM) are indicated by asterisks. **FigureS6**. Heat map of expression of genes involved incell cycle and ROS metabolism. (A) Graph of expression abundance of cellcycle-related genes. Differential expression profile of ROS synthesis (B) andclearance related genes (C). For transcriptome sequencing, selected uniformwheat plants after germination, half of which were transplanted into a nutrientsolution with normal Fe concentration for cultivation, and half weretransplanted into a nutrient solution with low Fe for cultivation, and sampleswere taken 10 days later. The heat map shows the gene expression levelindicated by the TPM value. Differentially expressed genes that show higher expressionlevels under he control (normal Fe: 50 μM) and treatment (low Fe: 2 μM) areindicated by asterisks. **Figure S7.** Co-expressionnetwork analysis of Fe absorption and transport related genes under low Festress conditions. Cycle nodes represent genes, and the size of the nodesrepresents the power of the interrelation among the nodes by degree value.Edges between two nodes represent interactions between genes. **Figure S8.** Natural variation in wheatresistance to low Fe stress and morphological identification of resistant andsensitive genotypes. (A) Variation in low Fe stress in a panel comprising 386wheat accessions. The root length and chlorophyll value of wheat were used forpopulation screening. After germination, uniform seedlings were selected andgrown for 10 d in Hoagland nutrient solution. Then Half of the seedlings werecultivated in a nutrient solution containing sufficient Fe, and half werecultivated in a nutrient solution containing low Fe stress. Min, minimum; max,maximum; CV, coefficient of variation. (B) Growth performance of ‘zhengmai1860’and ‘zhoumai32’ under low Fe stress. For the low Fe treatment, the wheat plantswere grown hydroponically under high Fe (50 μM) and low Fe (2 μM) conditionsfor 10 d, respectively. Bar: 5cm. (C) Relative expression of TaIRT1b-4A,TaNAS2-6D, TaNAS1a-6A, TaNAS1a-6B, and TaNAAT1b-1D in the shoots and roots ofthe two genotypes grown under normal Fe and low Fe conditions. For the low Fetreatment, the wheat plants were grown hydroponically under high Fe (50 μM) andlow Fe (2 μM) conditions for 10 d, respectively. Data are means (±SD), *n*=3. Thesignificant difference was determined using Student’s t-test: **P<0.01. **Table S2.** RT-qPCR primersequences.

## Data Availability

All data generated or analysed during this study are included in this published article [and its supplementary information files]. All the data and materials that are required to reproduce these findings can be shared by contacting Cai-peng Yue (yuecaipeng@zzu.edu.cn). All the sequencing data were submitted to the National Centre for Biotechnology Information (NCBI) (http://www.ncbi.nlm.nih.gov/) with the Bioproject of PRJNA744353.

## Abbreviations

BASS: Chloroplast-Localized Bile Acid
BP: Biological Process
CAO: Chlorophyll an Oxygenase
CBR: Chlorophyll B Reductase
CC: Cell Component
CCC: Ca^2+^-Sensitive Cross-Complementer
CS: Chlorophyll Synthase
CV: Coefficient of Variation
DAB: 3′3’-Diaminobenzidine
DEGs: Differentially Expressed Genes
DMA: Deoxysarcosine
DMAS: Deoxyergate Synthase
Fe: Iron
FIT: Fer-like Iron Deficiency-induced Transcription Factor
FPKM: Fragments Per Kilobase of Exon Model Per Million Mapped Reads
FPN: Ferroportin
FRO: Fe^3+^ Chelate Reductase
GGPP: Geranylgeranyl Diphosphate
GO: Gene Ontology
ICP-MS: Inductively Coupled Plasma Mass Spectrometry
IRT: Iron-Regulated Transporter
KEGG: Kyoto Encyclopedia of Genes and Genomes
LRP: Lateral Root Primordia
MATE: Multidrug and Toxin Efflux Family
MDA: Malondialdehyde
MF: Molecular Function
NAAT: Nicotinamide Aminotransferase
NAP: Non-intrinsic ABC protein
NAS: Nicotinamide Synthase
NBT: Nitro Blue Tetrazole
NCED: 9-*cis*-Epoxycarotenoid Dioxygenase
NRAMP: Natural Resistance-Associated Macrophage Protein
OFR: Superoxide Anion
OPT: Oligopeptide Transporter
PIC: Permease in Chioroplasts
Pro: Proline
PS: Phytosiderophore
PSY: Phytoene Synthase
QC: Quiescent Center
RBOH: Respiratory Burst Oxidase Homologous Gene
RIN: RNA Integrity Number
RT-qPCR: Reverse Transcription-quantitative Polymerase Chain Reaction
SAM: Methionine Synthetase
TOM: Transporter of Mugnetic Acid
TTC: Tetrazolium
VDE: Violaxanthin De-Epoxidase
VIT: Vacuolar Iron Transporter Ginseng
YSL: Yellow Stripe-Like
ZEP: Zeaxanthin Epoxidase
β-OHase: β-Carotene Hydroxylase
