# Supplementary material for: Combined morpho-physiological, ionomic and transcriptomic analyses reveal adaptive responses of allohexaploid wheat (Triticum aestivum L.) to iron deficiency
Source: BMC Plant Biol. 2022 May 10;22:234. doi: 10.1186/s12870-022-03627-4 (PMC9088122; doi:10.1186/s12870-022-03627-4)
Supplement: Supplementary file 2 — Additionalfile 2: Figure S1. Correlation analysis of shoot and root samples. HIR:High Iron Root, HIS: High Iron Shoot, LIR: Low Iron Root, LIS: Low Iron Shoot.1, 2, and 3 respectively represent different biological repetitions. Figure S2. Genomic distribution ofdifferentially expressed genes (DEGs). Genomic distribution of DEGs in the shoots (A)and roots (B). Figure S3. Molecularmodel of Fe absorption and transport related genes. A molecular model of genesinvolved in Fe absorption and transport in plant roots (A), chloroplasts,mitochondria, and vacuoles (B). FigureS4. Differential expression profiles of genes related to Fe absorption andtransport in wheat plants under low Fe stress. (A) Fe3+ chelatereductase (FRO), (B) naturalresistance-associated macrophage protein (NRAPM),(C) Yellow stripe-like (YSL), (D) methionine synthetase (SAM), (E) iron-regulated transporter (IRT), (F) Deoxyergate synthase (DMAS), (G) Nicotinamide aminotransferase(NAAT), (H) Mitochondrial m-typethioredoxin in chloroplast (ATM), (I)Nicotinamide synthase (NAS), (J)transporter of mugnetic acid (TOM),(K) multidrug and toxin efflux family (MATE),(L) Permease in chioroplasts (PIC),(M) Oligopeptide transporter (OPT),(N) mitoferrin, (O) mitoferrin-like, (P) Iron efflux transporter ferroportin (FPN), (Q) Nonintrinsic ABC protein (NAP), (R) vacuolar iron transporterginseng (VIT). For transcriptomesequencing, selected uniform wheat plants after germination, half of which weretransplanted into a nutrient solution with normal Fe concentration forcultivation, and half were transplanted into a nutrient solution with low Fefor cultivation, and samples were taken 10 days later. The heat map shows thegene expression level indicated by the TPM value. Differentially expressedgenes that show higher expression levels under the control (normal Fe: 50 μM)and treatment (low Fe: 2 μM) are indicated by asterisks. Figure S5. Differential expression profile ofphotosynthesis-related genes in wheat plants under low Fe str [file 12870_2022_3627_MOESM2_ESM.docx]

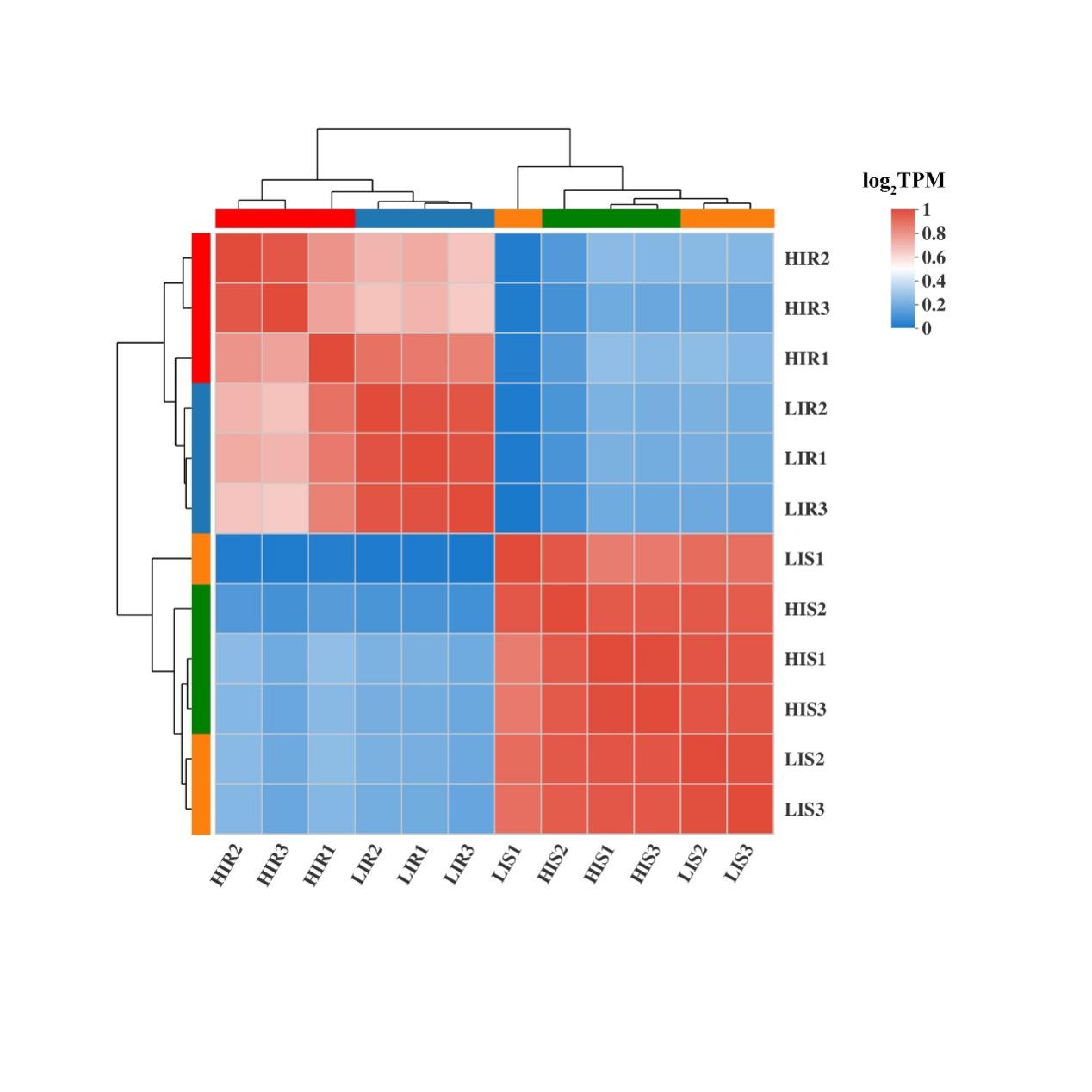


**Figure S1.** Correlation analysis of shoot and root samples. HIR: High Iron Root, HIS: High Iron Shoot, LIR: Low Iron Root, LIS: Low Iron Shoot. 1, 2, and 3 respectively represent different biological repetitions.


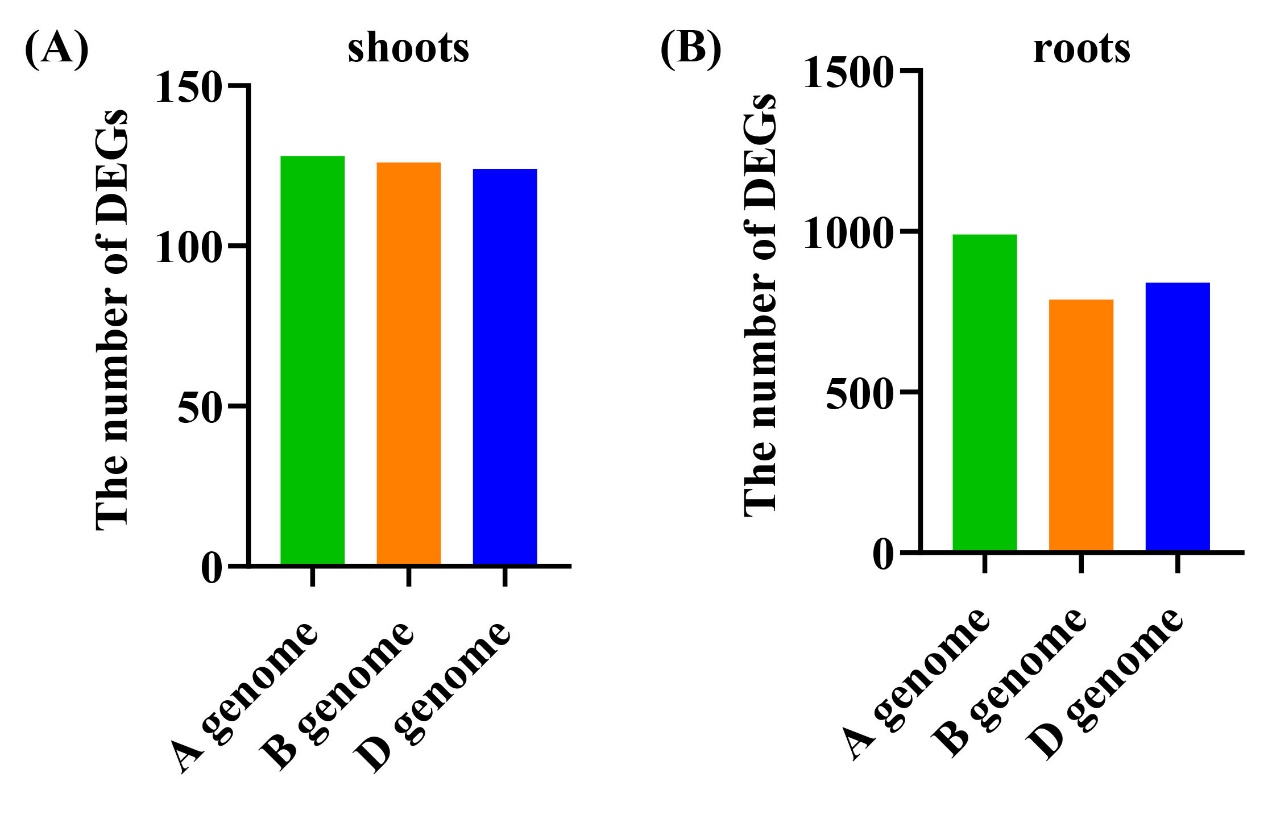
**Figure S2.** Genomic distribution of differentially expressed genes (DEGs). Genomic distribution of DEGs in the shoots (A) and roots (B).


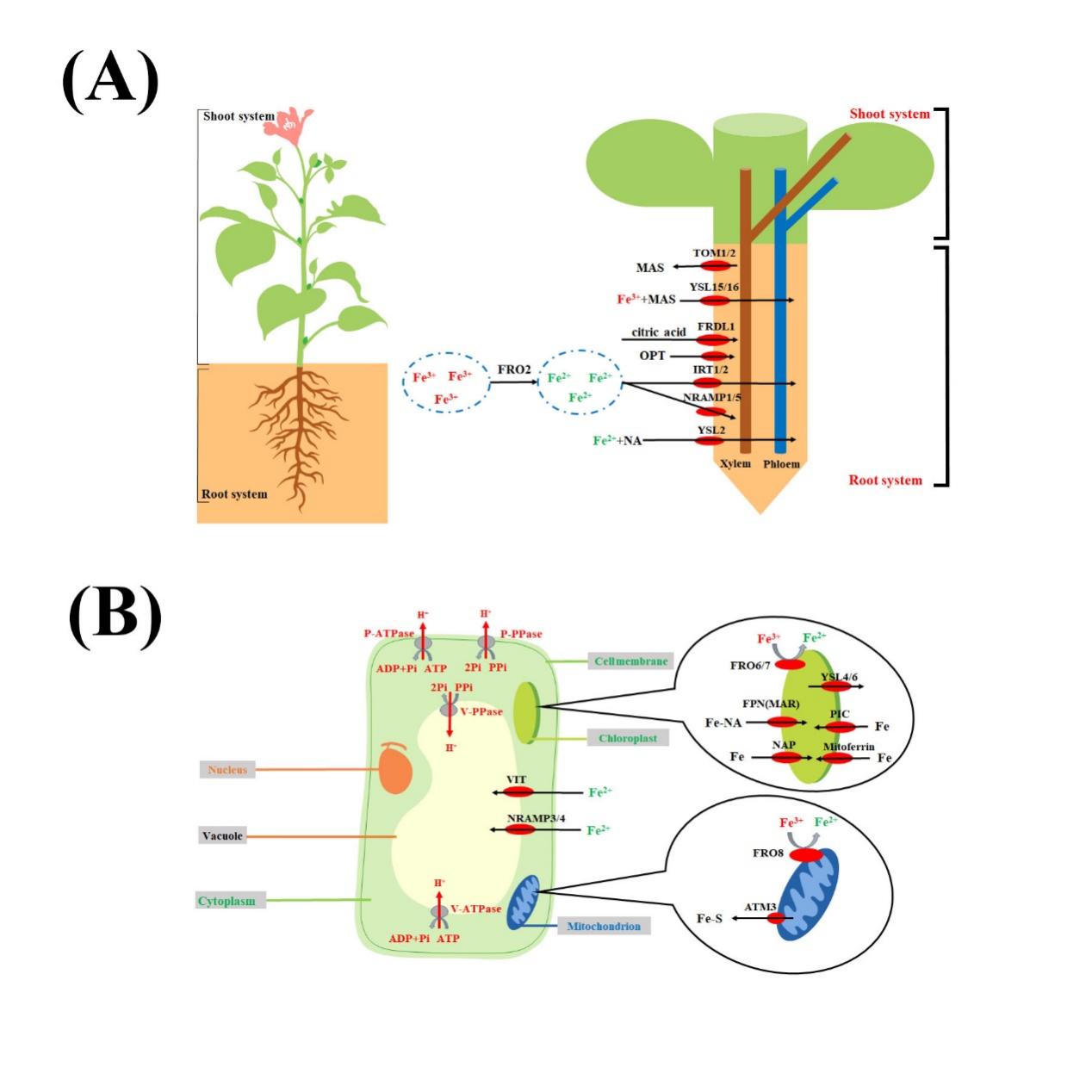


**Figure S3** Molecular model of Fe absorption and transport related genes. A molecular model of genes involved in Fe absorption and transport in plant roots (A), chloroplasts, mitochondria, and vacuoles (B).


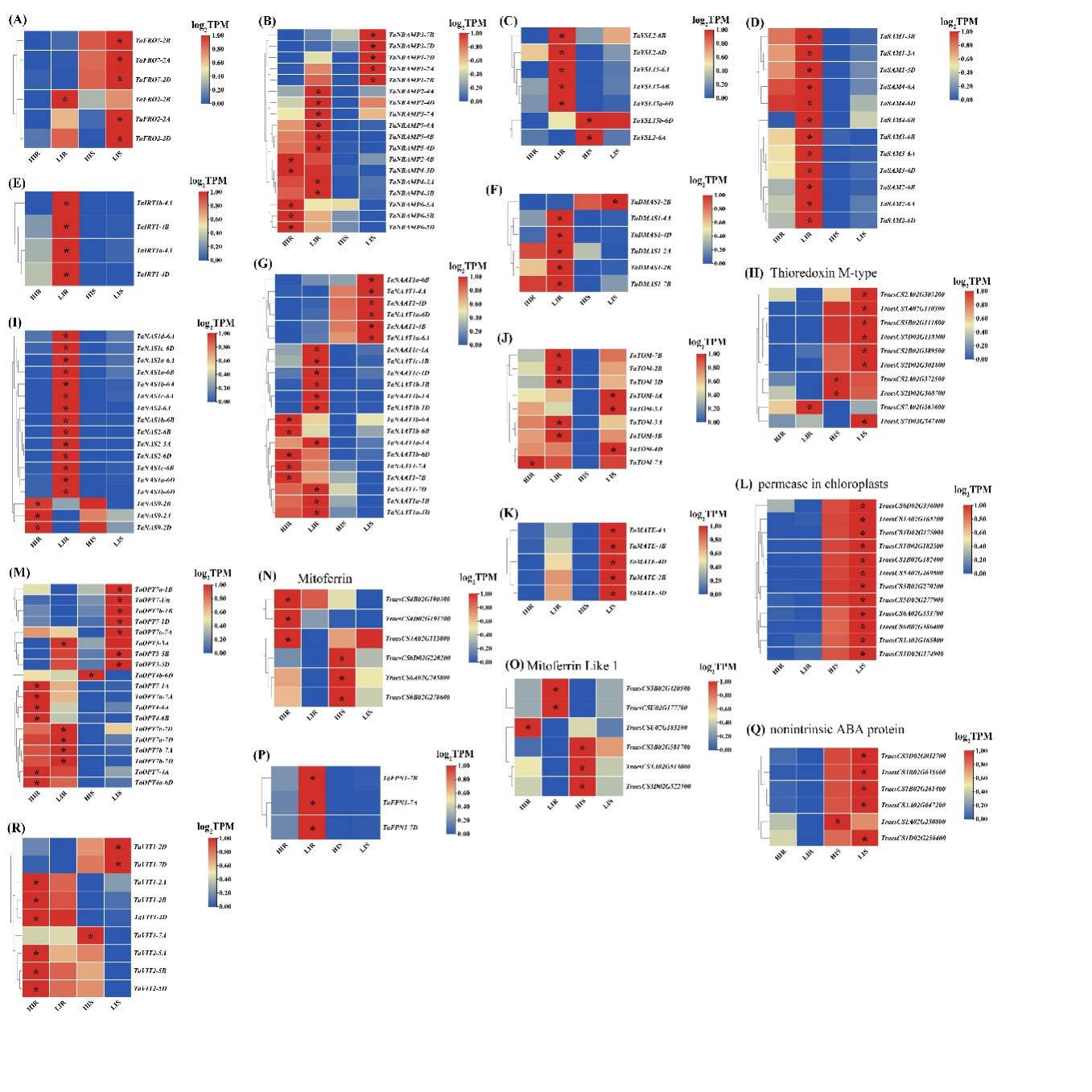
**Figure S4** Differential expression profiles of genes related to Fe absorption and transport in wheat plants under low Fe stress. (A) Fe^3+^ chelate reductase (*FRO*), (B) natural resistance-associated macrophage protein (*NRAPM*), (C) Yellow stripe-like (*YSL*), (D) methionine synthetase (*SAM*), (E) iron-regulated transporter (*IRT*), (F) Deoxyergate synthase (*DMAS*), (G) Nicotinamide aminotransferase (*NAAT*), (H) Mitochondrial m-type thioredoxin in chloroplast (*ATM*), (I) Nicotinamide synthase (*NAS*), (J) transporter of mugnetic acid (*TOM*), (K) multidrug and toxin efflux family (*MATE*), (L) Permease in chioroplasts (*PIC*), (M) Oligopeptide transporter (*OPT*), (N) mitoferrin, (O) mitoferrin-like, (P) Iron efflux transporter ferroportin (*FPN*), (Q) Nonintrinsic ABC protein (*NAP*), (R) vacuolar iron transporter ginseng (*VIT*). For transcriptome sequencing, selected uniform wheat plants after germination, half of which were transplanted into a nutrient solution with normal Fe concentration for cultivation, and half were transplanted into a nutrient solution with low Fe for cultivation, and samples were taken 10 days later. The heat map shows the gene expression level indicated by the TPM value. Differentially expressed genes that show higher expression levels under the control (normal Fe: 50 μM) and treatment (low Fe: 2 μM) are indicated by asterisks.


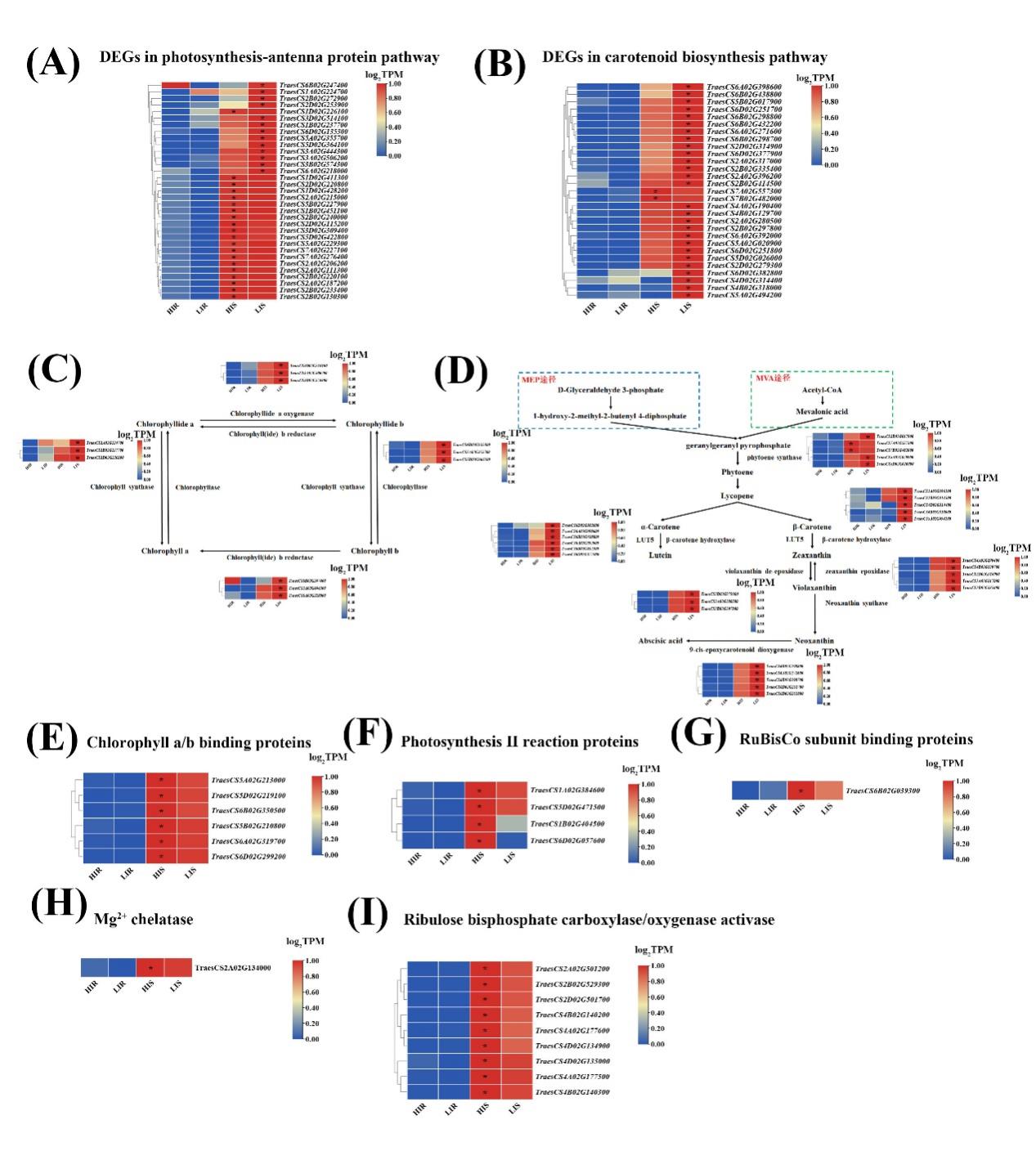


**Figure S5** Differential expression profile of photosynthesis-related genes in wheat plants under low Fe stress. Differential expression profile of genes related to photosynthesis-antenna proteins (A) and carotenoid biosynthesis (B). The expression pattern of genes involved in the transformation of chlorophyll a and chlorophyll b (C) and the synthesis of carotenoids under low Fe stress (D). The expression pattern of chlorophyll a/b binding proteins (E), Photosynthesis II reaction proteins (F), RuBisCo subunit binding proteins (G), Mg^2+^ chelatase (H), Ribulose bisphosphate carboxylase/oxygenase activase (I). For transcriptome sequencing, selected uniform wheat plants after germination, half of which were transplanted into a nutrient solution with normal Fe concentration for cultivation, and half were transplanted into a nutrient solution with low Fe for cultivation, and samples were taken 10 days later. The heat map shows the gene expression level indicated by the TPM value. Differentially expressed genes that show higher expression levels under he control (normal Fe: 50 μM) and treatment (low Fe: 2 μM) are indicated by asterisks.


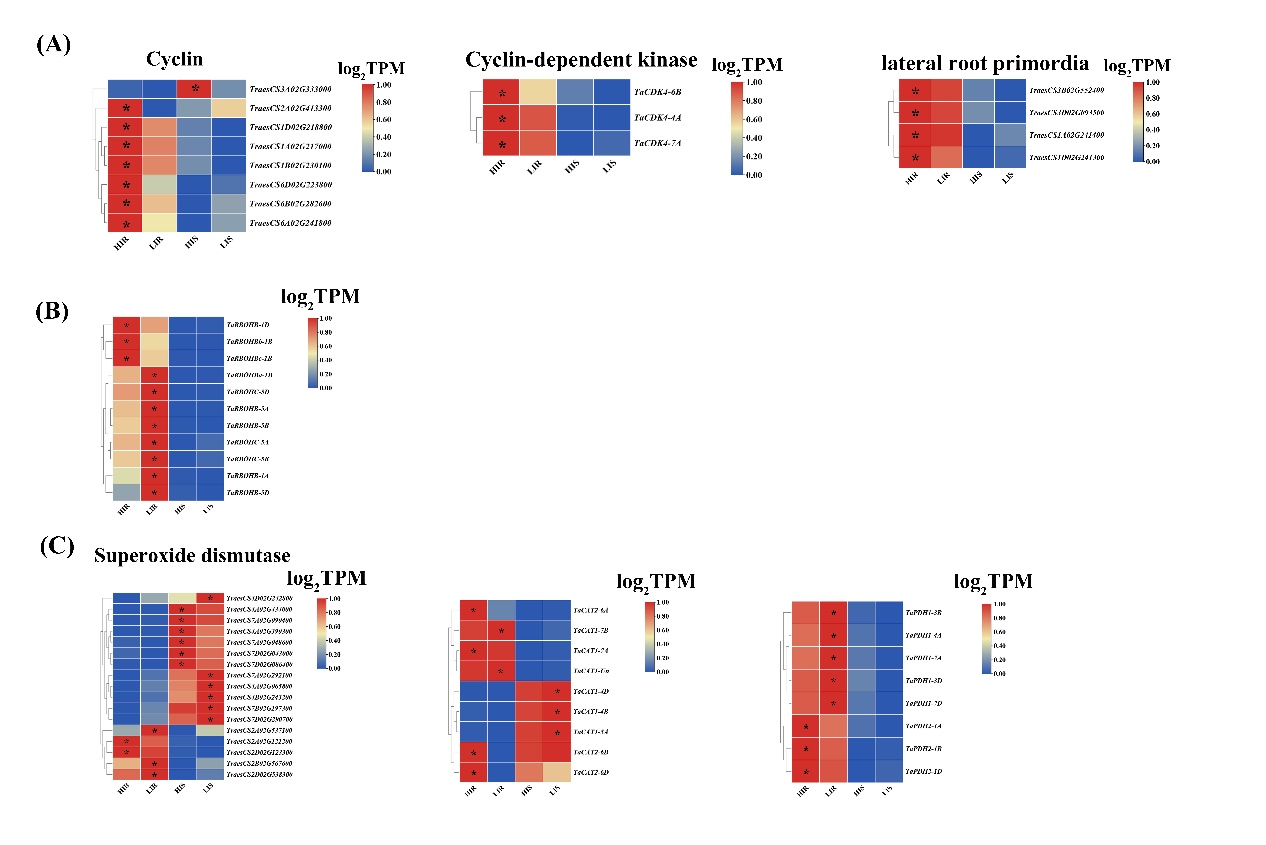


**Figure S6** Heat map of expression of genes involved in cell cycle and ROS metabolism. (A) Graph of expression abundance of cell cycle-related genes. Differential expression profile of ROS synthesis (B) and clearance related genes (C). For transcriptome sequencing, selected uniform wheat plants after germination, half of which were transplanted into a nutrient solution with normal Fe concentration for cultivation, and half were transplanted into a nutrient solution with low Fe for cultivation, and samples were taken 10 days later. The heat map shows the gene expression level indicated by the TPM value. Differentially expressed genes that show higher expression levels under he control (normal Fe: 50 μM) and treatment (low Fe: 2 μM) are indicated by asterisks.


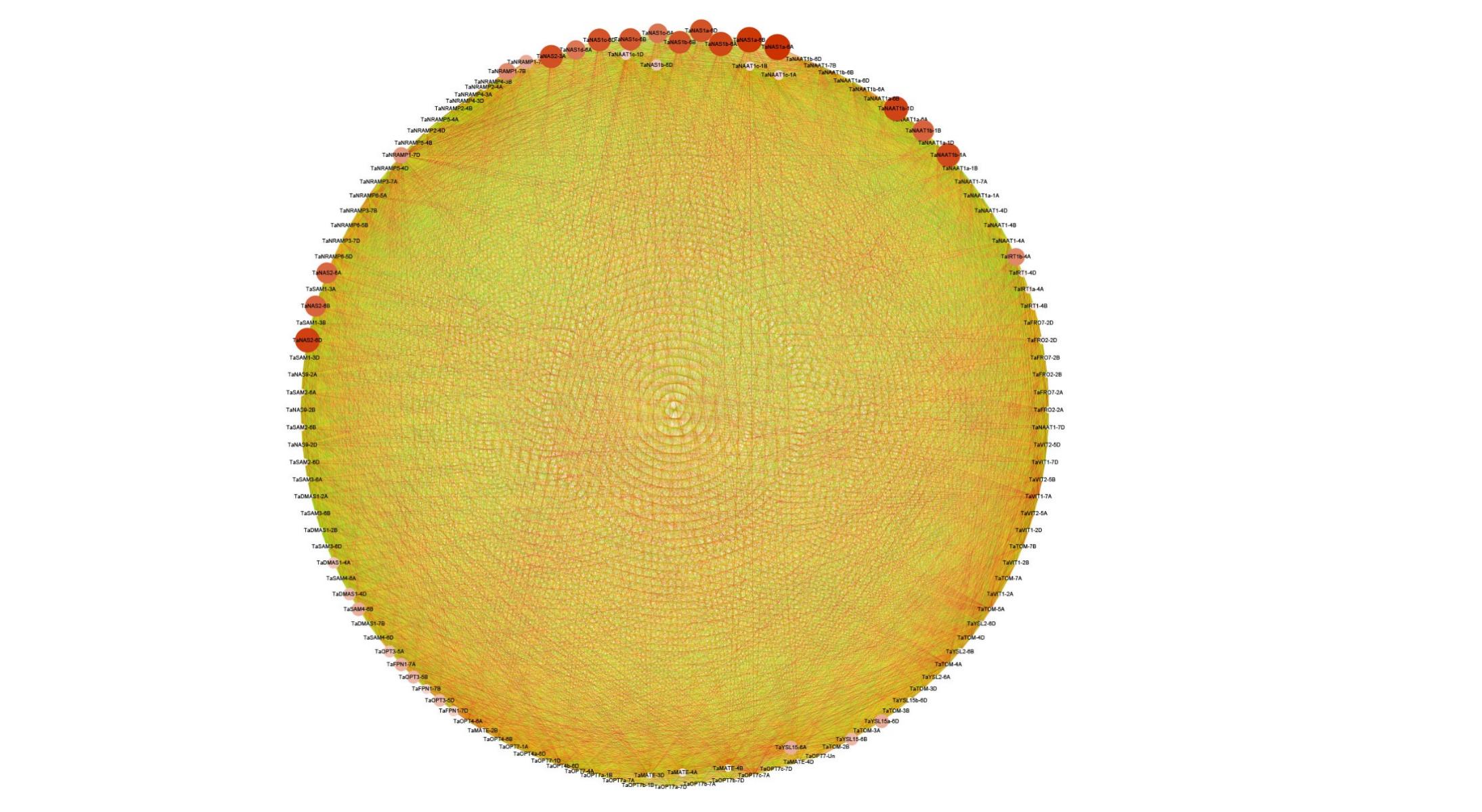
**Figure S7** Co-expression network analysis of Fe absorption and transport related genes under low Fe stress conditions. Cycle nodes represent genes, and the size of the nodes represents the power of the interrelation among the nodes by degree value. Edges between two nodes represent interactions between genes.


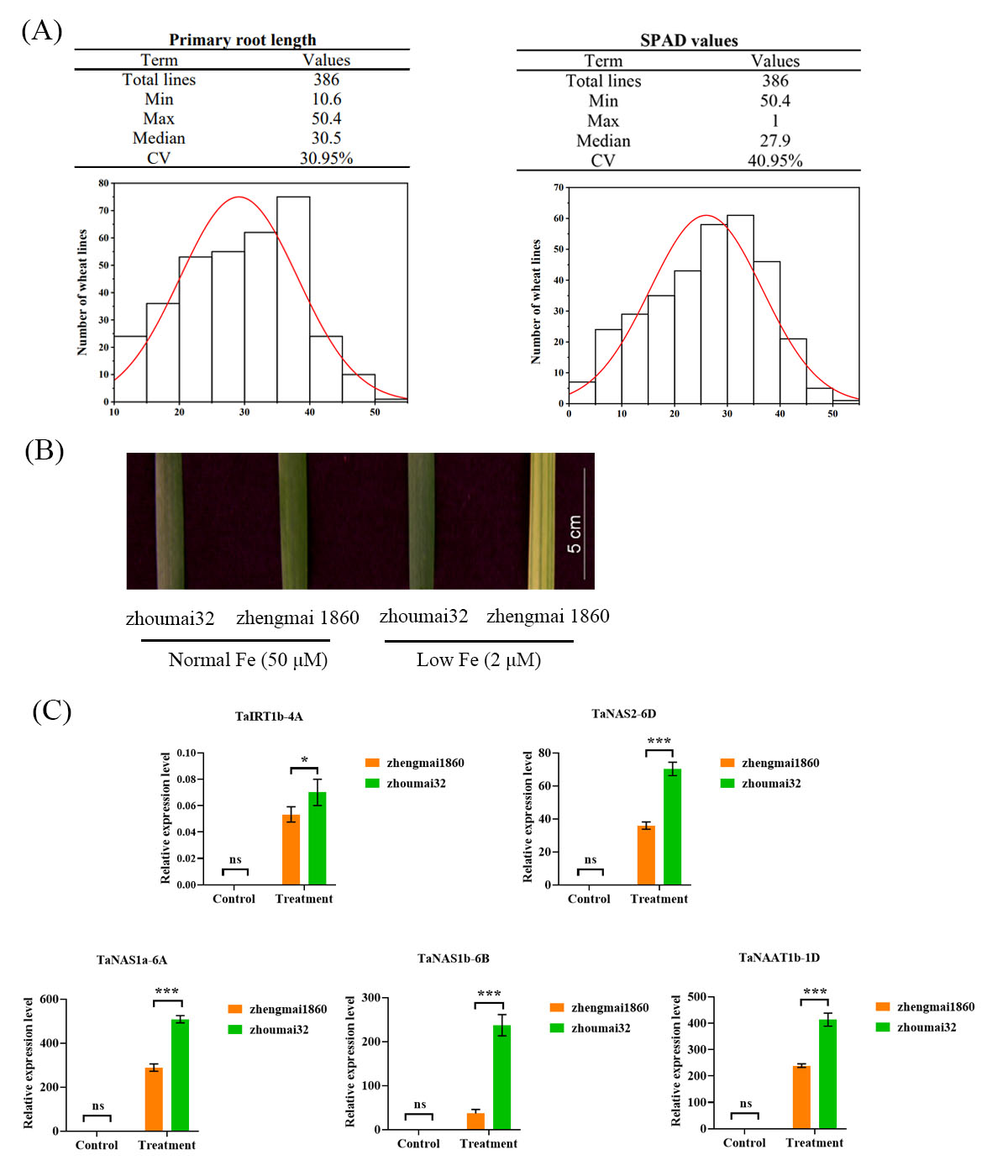
**Figure S8** Natural variation in wheat resistance to low Fe stress and morphological identification of resistant and sensitive genotypes. (A) Variation in low Fe stress in a panel comprising 386 wheat accessions. The root length and chlorophyll value of wheat were used for population screening. After germination, uniform seedlings were selected and grown for 10 d in Hoagland nutrient solution. Then Half of the seedlings were cultivated in a nutrient solution containing sufficient Fe, and half were cultivated in a nutrient solution containing low Fe stress. Min, minimum; max, maximum; CV, coefficient of variation. (B) Growth performance of ‘zhengmai1860’ and ‘zhoumai32’ under low Fe stress. For the low Fe treatment, the wheat plants were grown hydroponically under high Fe (50 μM) and low Fe (2 μM) conditions for 10 d, respectively. Bar: 5cm. (C) Relative expression of TaIRT1b-4A, TaNAS2-6D, TaNAS1a-6A, TaNAS1a-6B, and TaNAAT1b-1D in the shoots and roots of the two genotypes grown under normal Fe and low Fe conditions. For the low Fe treatment, the wheat plants were grown hydroponically under high Fe (50 μM) and low Fe (2 μM) conditions for 10 d, respectively. Data are means (±SD), n=3. The significant difference was determined using Student’s t-test: **P<0.01.

| **Table S2** RT-qPCR primer sequences | |
| --- | --- |
| Gene | Sequences (5‘-3’) |
| *TaGAPDH-F* | AAGGCTGTTGGCAAGGTG |
| *TaGAPDH-R* | GTGGTCGTTCAGAGCAATCC |
| *TaIRT1b-4A-F* | CCACACATGCTCGAGCAAT |
| *TaIRT1b-4A-R* | CAACCTCAGCTCGCCGTGCC |
| *TaNAS2-6D-F* | GAACTCGAGTACGATTGAAG |
| *TaNAS2-6D-R* | GATTCTCCTCCCCATCAC |
| *TaNAS1a-6A-F* | GAAAGGAACCGAAATGCAT |
| *TaNAS1a-6A-R* | CTTATCATCGACATCTC |
| *TaNAS1b-6B-F* | GATACATTTCAGCGACTTC |
| *TaNAS1b-6B-R* | TCCAAAAGAATTTTCAACTT |
| *TaNAAT1b-1D-F* | CGGAAGCCCCAGTTCATGTGC |
| *TaNAAT1b-1D-R* | GCATTTACACACACGATTTC |
